# Machine Learning-Assisted Development of Injectable, Mechanically Robust, and Energy Metabolism-Modulating Brushite Cements

**DOI:** 10.34133/research.0776

**Published:** 2025-07-10

**Authors:** Dachuan Liu, Jiaxu Shi, Youhao Ni, Li Dong, Chen Cui, Lijie Wang, Yu Zhang, Jingxi Xu, Weicheng Chen, Kai Lu, Miodrag J. Lukic, Wei Xia, Song Chen, Bin Li

**Affiliations:** ^1^ Orthopedic Institute, Department of Orthopedic Surgery, Medical 3D Printing Center, The First Affiliated Hospital, MOE Key Laboratory of Geriatric Diseases and Immunology, School of Basic Medical Sciences, Suzhou Medical College, Soochow University, Suzhou, Jiangsu 215000, China.; ^2^ Key Laboratory of C&PC Structures of Ministry of Education, Southeast University, Nanjing 211189, China.; ^3^ Sanitation & Environment Technology Institute of Soochow University Ltd., Suzhou, Jiangsu 215000, China.; ^4^ Department of Orthopedics, Northern Jiangsu People’s Hospital Affiliated to Yangzhou University, Yangzhou 225001, China.; ^5^ Laboratory of Physics, “Vinca” Institute of Nuclear Sciences, National Institute of the Republic of Serbia, University of Belgrade, Belgrade, Serbia.; ^6^ Applied Materials Science, Department of Engineering Science, Uppsala University, Uppsala, Sweden.

## Abstract

In orthopedic minimally invasive surgeries (MIS) such as percutaneous vertebroplasty (PVP) and percutaneous kyphoplasty (PKP), calcium phosphate cements (CPCs) are an attractive alternative to bioinert polymethyl methacrylate (PMMA) due to their superior biocompatibility and osteoconductivity. However, the mechanical strength and injectability of CPCs often remain insufficient for load-bearing applications, limiting their broader use in these critical procedures. To address this challenge, we introduce a machine learning-assisted approach to enhance both the mechanical strength and injectability of CPCs by identifying specific polymers as superplasticizers. By optimizing its concentration and the liquid-to-powder (L/P) ratio, we developed an injectable brushite-based cement with an exceptional compressive strength of 79.5 ± 4.3 MPa, surpassing both traditional CPCs and PMMA in orthopedic applications. Zeta potential and adsorption studies reveal that these superplasticizers enhance cement paste dispersion via electrostatic repulsion. In vitro assays demonstrate excellent biocompatibility and osteogenic properties, while in vivo experiments further confirm the cement’s superior osteoinductive capability. The brushite cement regulates cellular metabolism and stem cell differentiation by enhancing energy metabolism and activating key signaling pathways such as phosphatidylinositol 3-kinase–AKT and mitogen-activated protein kinase–extracellular signal–regulated kinase. These findings offer a novel approach to fabricating CPCs with enhanced mechanical strength and osteogenic potential, addressing long-standing challenges in orthopedic MIS.

## Introduction

Calcium phosphate cements (CPCs) are widely used as bone void fillers in orthopedics and dentistry due to their excellent biocompatibility and osteoconductivity [1–3]. Recently, CPCs have been proposed as an alternative to polymethyl methacrylate (PMMA) for minimally invasive surgery (MIS), such as percutaneous vertebroplasty (PVP) and percutaneous kyphoplasty (PKP). However, CPCs are generally weak and exhibit poor injectability, limiting their applications in PVP and PKP procedures [4]. Strategies to strengthen CPCs focus on reducing porosity or enhancing the inorganic network of cements. For example, the compressive strength of setting cements was increased by compaction, i.e., via reduced porosity [5,6]. However, this strategy cannot be employed in MISs since no pre-pressure can be applied after injecting the paste into injured sites. Using a low liquid-to-powder (L/P) ratio yields cements with higher mechanical strength but decreases injectability and workability [7]. Ionic substitutions [8–11] and the formation of an interpenetrating network within the cement matrix have also proven effective in strengthening CPCs [12,13]. Yet, the benefits of these strategies are rather limited, and in some cases, they may negatively impact degradation and biological performance [14,15]. Therefore, new approaches need to be explored to enhance the applicability of CPCs in MIS, specifically in load-bearing applications.

The invention of superplasticizers represents one of the major breakthroughs and milestones in the history of concrete [16]. Superplasticizers play an essential role in developing concrete with high strength [17]. They have been utilized to improve the workability of concrete under the same water/binder ratio or enhance the compressive strength while keeping the same workability [18]. To date, various superplasticizers, such as polycarboxylate ethers (PCEs), poly-naphthalene sulfonate (PNS), and lignosulfonate (LS), were synthesized and successfully used in the civil engineering field [19–21]. Despite advancements in the development of superplasticizers, studies on superplasticizers for CPCs are quite limited. Based on the final set of products, CPCs primarily fall into 2 categories: brushite cement and apatite cement. Both types of cements harden through a dissolution–recrystallization process similar to that of Portland cements [22,23]. Theoretically, certain polymers could act as superplasticizers for CPCs, potentially leading to the fabrication of CPCs with enhanced performance. Fernández et al. [24] studied the effect of commercial superplasticizers, commonly used for Portland cement, on apatite cement, showing that these additives markedly reduced the water content but increased the compressive strength. However, the exact components and water-reducing mechanisms of these superplasticizers, which are typically mixtures of copolymers, have remained unknown. Notably, despite the great potential of brushite cements, known for their biodegradability and osteogenic properties, there are no reports on superplasticizers specifically designed for them [25,26]. In a previous study, we developed a high-strength brushite cement using ammonium iron citrate (AIC) solution as the liquid phase [27], setting the next goal to explore the possibility of fabricating high-performance AIC-brushite cements with enhanced injectability and compressive strength by using specific superplasticizers.

Recently, machine learning (ML) has emerged as a promising technique for addressing diverse scientific challenges and finding optimal solutions [28–33]. ML has been extensively applied across various fields, with numerous algorithms developed to predict materials’ compressive strength. For instance, Chithra et al. [34] effectively utilized an artificial neural network (ANN) algorithm to predict the compressive strength of high-performance concrete incorporating nano-silica and copper slag. Similarly, Zhang et al. [35] developed 12 distinct ML-based regressors to forecast concrete compressive strength, demonstrating the superior accuracy of a deep forest-based model. Additionally, Aslam et al. [36] created a gene expression programming (GEP)-based model to predict the compressive strength of high-strength concrete using a substantial dataset comprising 357 data points. The use of ML for screening suitable superplasticizers may provide a crucial approach for constructing and optimizing high-strength bone cements.

In addition to the injectability and mechanical strength, osteogenic properties are essential for the clinical performance of CPCs. Following a bone injury, e.g., fracture, the bone microenvironment and blood vessels are compromised, leading to increased inflammation and local hypoxia [37,38]. Under these conditions, bone marrow mesenchymal stem cells (BMSCs) often resort to anaerobic glycolysis, as the tricarboxylic acid (TCA) cycle and oxidative phosphorylation (OxPhos) become impaired, leading to nonunion or delayed fracture union [39,40]. The TCA cycle is critical for energy production, providing the adenosine triphosphate (ATP) necessary for various biological activities [41–43]. Citrate, a setting retardant for brushite cement, is a metabolite formed in the TCA cycle, and it serves as an essential metabolic intermediate and pivotal regulator of energy production [44]. Several studies have demonstrated that the addition of citrate positively influences the healing of bone defects [45] and stimulate bone tissue healing by up-regulating the membrane transporters, specifically SLC13a5 [43,46]. Moreover, citrates prevent osteoporosis and enhance the density and number of bone trabeculae [47,48]. Therefore, the presence of citrates in brushite cement may improve energy metabolism at the bone injury site, effectively promoting bone repair.

**Fig. 1.**
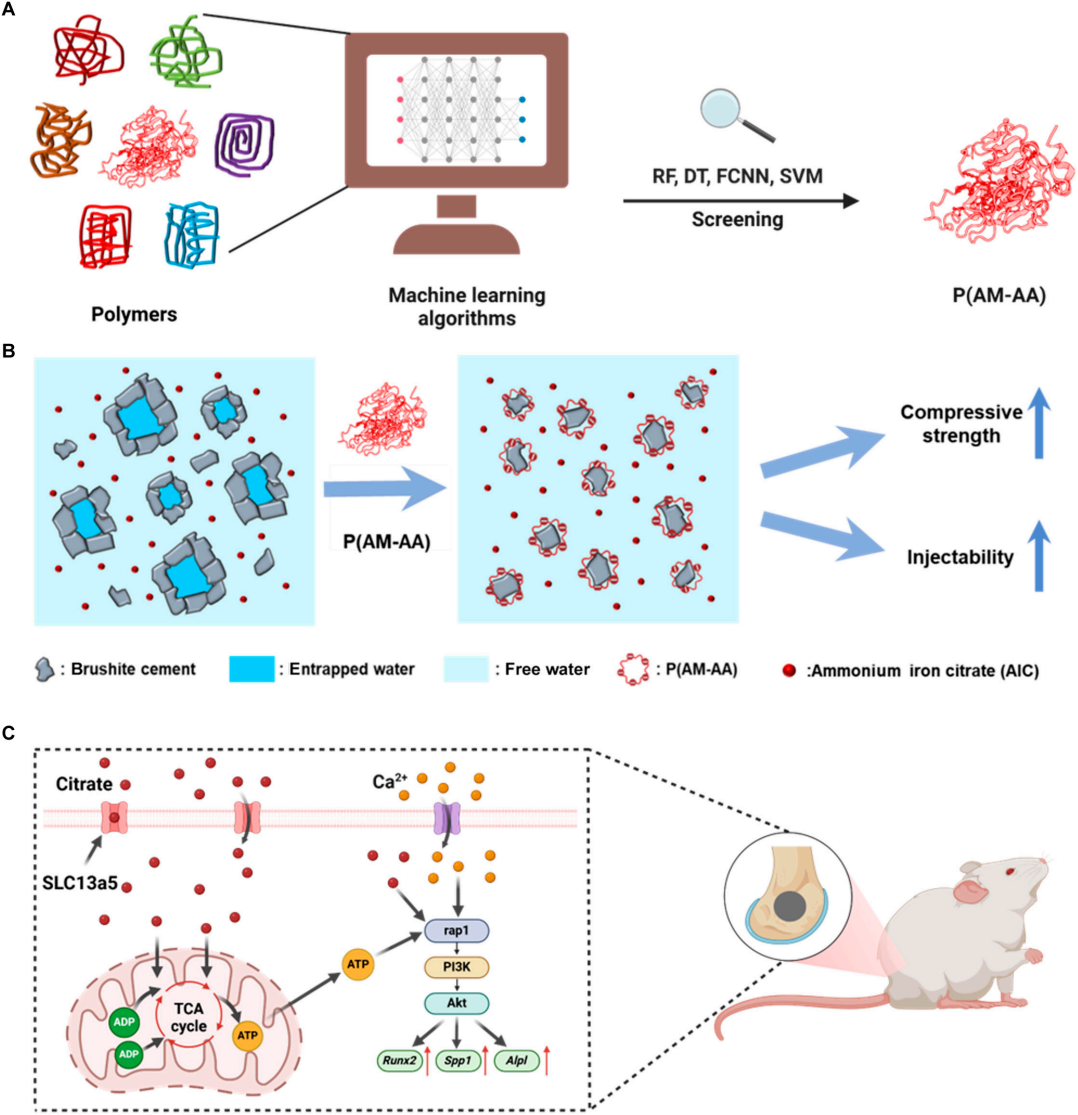
Schematic illustration of high-performance calcium phosphate cements engineered with superplasticizers for application in bone repair. (A) Screening of superplasticizers using machine learning. (B) Water-reducing mechanism of the P(AM-AA) superplasticizer. (C) Application of the prepared high-performance brushite cement for femoral bone repair. RF, random forest; DT, decision tree; FCNN, full connect neural network; SVM, support vector machine; P(AM-AA), poly (acrylamide-co-acrylic acid); AIC, ammonium iron citrate; TCA, tricarboxylic acid cycle.

In this study, we explored a novel approach of utilizing superplasticizers to fabricate high-performance brushite cement, assisted by ML (Fig. 1). Initially, we screened for effective superplasticizers for brushite and apatite cements using ML techniques. The formulation of brushite cement is based on our previous study, in which AIC was used as the setting retarder [27]. The tested chemicals were poly (acrylamide-co-acrylic acid) [P(AM-AA)], acrylic acid-2-hydroxypropyl acrylate copolymer (AA/HPA), poly(acrylic acid-co-maleic acid) [P(AA-MA)], acrylic acid-2-acrylamido-2-methylpropyl sulfonic acid copolymer (AA/AMPS), polyglutamic acid (PGA), sodium ligninsulfonate (SL), (2-naphthalenesulfonic acid, polymer with formaldehyde, sodium salt) (PSA), and *N*-(tris(hydroxymethyl)methyl)-2-aminoethanesulfonic acid sodium (TES). By using poly(acrylamide-co-acrylic acid) [P(AM-AA)] as the superplasticizer, brushite cement with compressive strength (79.5 ± 4.3 MPa) comparable to those of nondegradable polymeric materials was prepared. Furthermore, we investigated the biocompatibility and osteogenic potential of 3 commonly used bone cements: brushite (with AIC and superplasticizer), apatite, and PMMA cements. We verified, by detecting ATP, NADH [reduced form of nicotinamide adenine dinucleotide (oxidized form)], and RNA sequencing, that citrates, used as a setting retarder, enhanced energy metabolism processes, promoted ATP production, and accelerated osteogenesis. Finally, we examined the osseointegration and regeneration capacity of the synthesized brushite cement (with AIC and superplasticizer) in vivo using micro-computed tomography (CT), histological staining, and immunofluorescent (IF) staining.

## Results and Discussion

### Prediction and evaluation of ML models

**Fig. 2.**
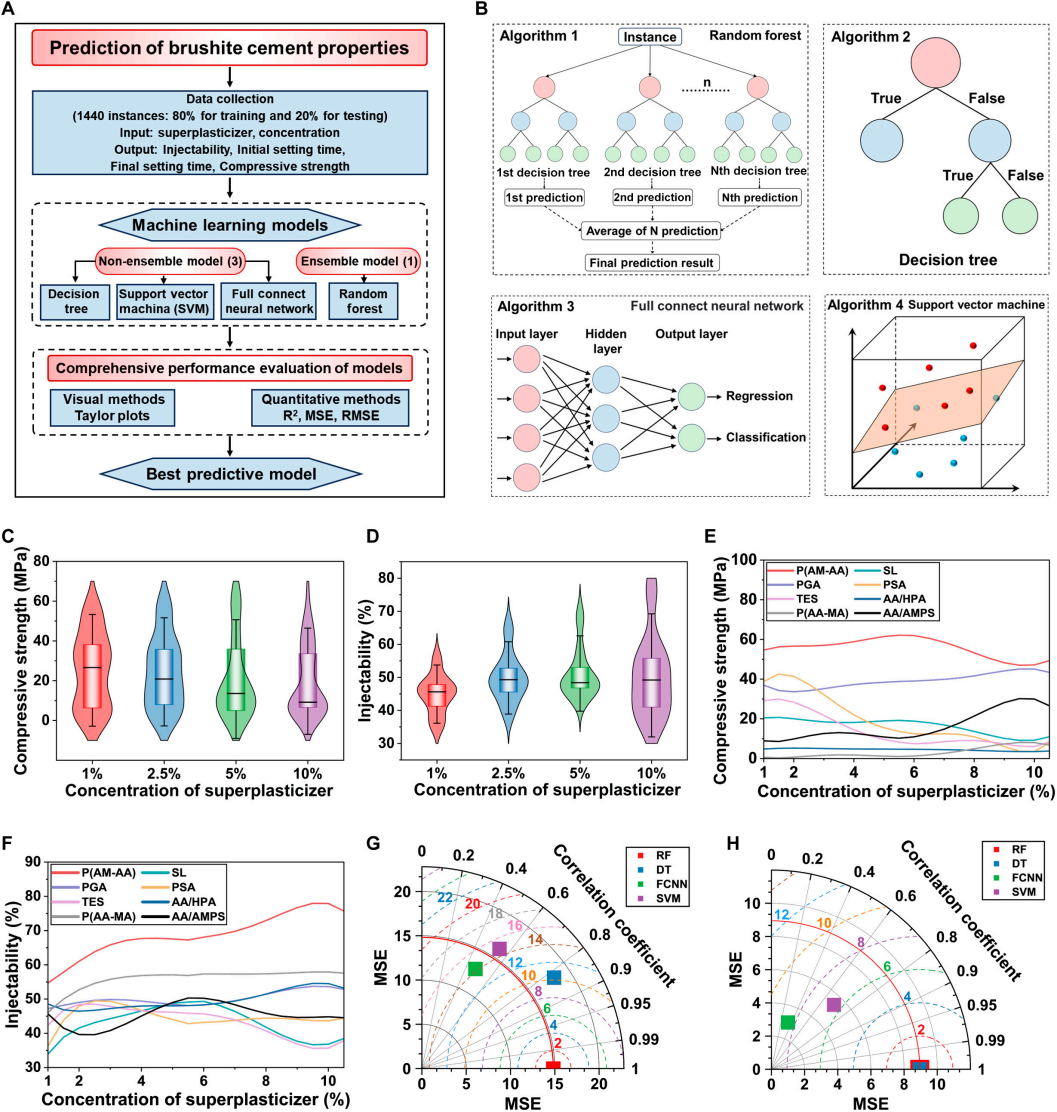
Machine learning (ML) for the screening and prediction of superplasticizers. (A) ML methodology flowchart. (B) Pictorial representation of ML models. (C and D) Violin plots illustrating the collected experimental data. (E and F) Prediction of compressive strength (E) and injectability (F) of brushite cement influenced by the type and concentration of superplasticizers using the RF model. (G and H) Taylor diagrams indicating the error metrics of DT models on compressive strength (G) and injectability (H) of brushite cements modified by different superplasticizers. [The ideal values (*R*^2^, MSE, RMSE) for these indices are as follows: *R*^2^ should ideally be 1, indicating perfect prediction accuracy, while RMSE and MSE should ideally be 0, indicating no error in the predictions.]

Injectability, compressive strength, and setting times are critical factors for the successful application of bone cements in MIS. In a previous study, we developed an injectable and biodegradable high-strength iron-bearing brushite cement [27]. This study explores the impact of superplasticizers on the injectability, compressive strength, and setting times of brushite cement, employing ML techniques to optimize the experimental outcomes. Eight chemicals, which could potentially serve as superplasticizers for CPCs, have been investigated: P(AM-AA), AA/HPA, P(AA-MA), AA/AMPS, PGA, SL, PSA, and TES (Fig. S1). β-Tricalcium phosphate (β-TCP) and monocalcium phosphate monohydrate (MCPM) mixed in a weight ratio of 1:1 served as the powder phase, and 0.55 M AIC and different concentrations of superplasticizers served as the liquid phase. The injectability (Figs. S2 and S3), compressive strength (Figs. S4 and S5), and setting time (Figs. S6 and S7) of the cements with various superplasticizers were measured. ML, a proven technique for estimating, classifying, and predicting materials’ strength based on varying material properties [49], was used to analyze the measured data. Figure 2A shows a flowchart of the methodology applied for predicting injectability, compressive strength, and setting time. The sample volume and the efficiency and accuracy of model prediction were comprehensively considered in the selection of ML models. Four ML models were applied for these predictions: algorithm 1, random forest (RF); algorithm 2, decision tree (DT); algorithm 3, full connect neural network (FCNN); and algorithm 4, support vector machine (SVM) (Fig. 2B). The compressive strength and injectability data distributions obtained for all investigated superplasticizers were non-normal, as depicted by the violin plots in Fig. 2C and D, respectively, for different concentrations of superplasticizers [the values of compressive strength (Fig. 2C) and injectability (Fig. 2D) of brushite cement are plotted for each specified superplasticizer concentration, i.e., 4 violin data plots in each figure panel correspond to 4 studied superplasticizer concentrations]. The data were input into 4 ML models to predict the changes in the properties of brushite cement in the presence of different superplasticizers: For the RF model, the predicted compressive strength (Fig. 2E), injectability (Fig. 2F), and setting time (Fig. S8) are shown, while the data for other ML models are shown in Figs. S9 to S11. Among these models, FCNN demonstrated the lowest mean squared error (MSE), indicating the superior performance among the applied models, as evaluated by the Taylor diagram (Fig. 2G and H and Fig. S12). The RF model exhibited the highest *R*^2^ value, indicating highest prediction accuracy. According to the RF model results, polymeric P(AM-AA) significantly improved the compressive strength of brushite cement while maintaining high injectability and short setting time (Fig. 2E and F and Fig. S8). Therefore, P(AM-AA) was selected as the superplasticizer in subsequent experiments.

### Characterization of CPCs with superplasticizers

**Fig. 3.**
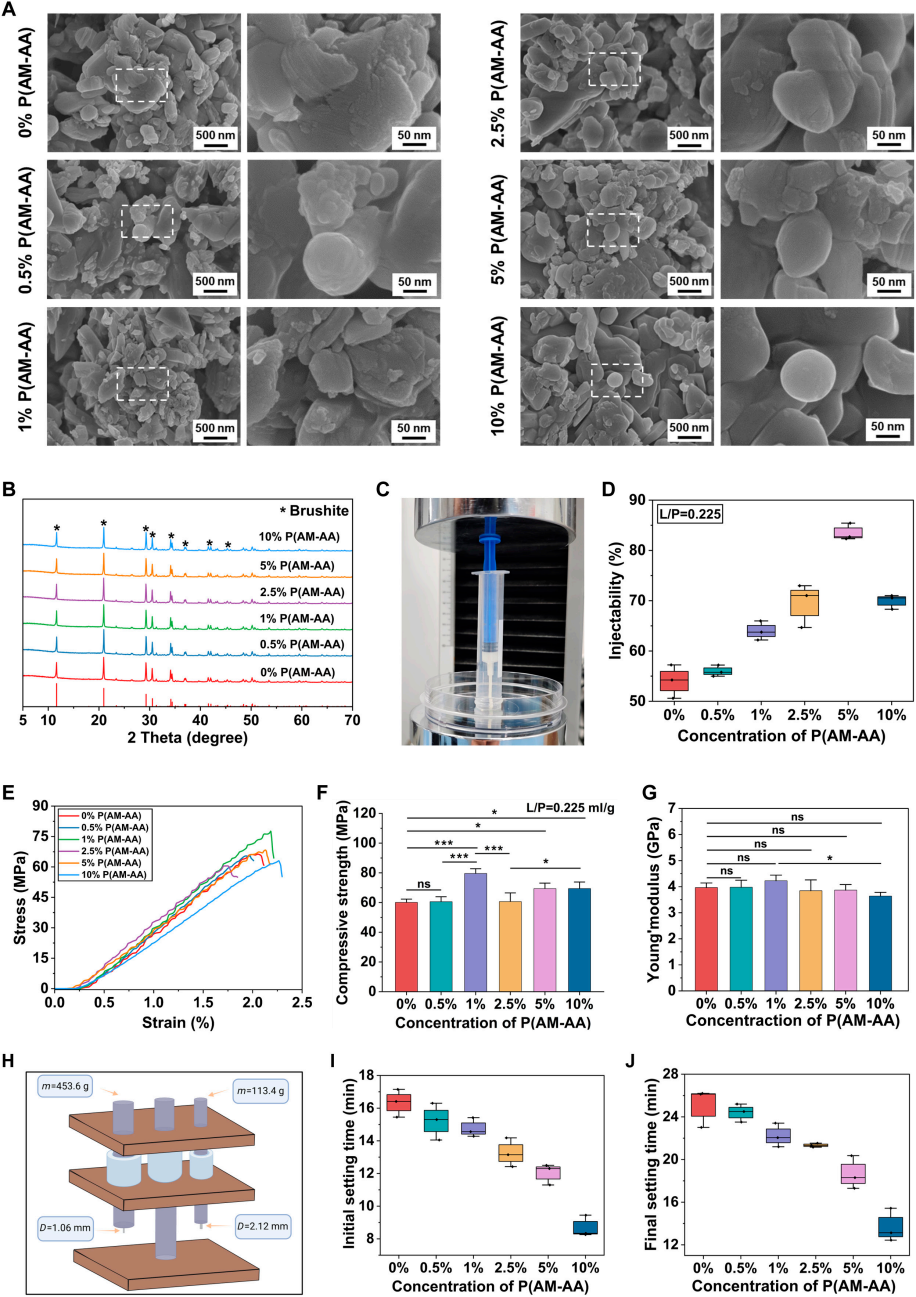
Characterization of brushite cements (0.14 M AIC). (A) Scanning electron microscopy (SEM) images of brushite cements with different P(AM-AA) concentrations. (B) XRD patterns of brushite cements hydrated in PBS for 24 h. (C) Schematic illustration depicting the injectability of the cements. (D) Injectability of brushite cements with different P(AM-AA) concentrations. Mechanical properties of brushite cements including stress–strain curves (E), compressive strength (F), and Young’s modulus (G). (H) Schematic illustration of the Gillmore needle used for measuring the setting time. (I and J) Initial setting time (I) and final setting time (J) of brushite cements. The error bars represent the SD obtained by 3 independent repeated measurements. Statistical analysis was performed using one-way ANOVA, with significance defined as *P < 0.05, **P < 0.01, ***P < 0.001.

In our previous study, we demonstrated the effect of AIC concentration on the compressive strength of brushite cement [27]. We have now further validated this effect in the presence of P(AM-AA) at an L/P ratio of 0.25 ml/g (Fig. S13 shows the influence of AIC concentration of the compressive strength of brushite cement without superplasticizer). Additionally, various properties of brushite cement (0.26 M AIC) with varying amounts of P(AM-AA) were further characterized. The set products [hydrated in phosphate-buffered saline (PBS) for 24 h] exhibited irregular morphology, and they consisted of pure brushite regardless of the P(AM-AA) concentration (Fig. 3A and B). To further improve the compressive strength of brushite cements, a lower L/P ratio of 0.225 ml/g was selected in later experiments (Fig. 3C and Fig. S14A and B). The addition of P(AM-AA) significantly increased the compressive strength and injectability of the cements. The highest injectability and compressive strength reached up to 83.5% and 79.5 MPa, respectively (Fig. 3D to F), where the latter is close to the theoretical compressive strength of brushite cement (83 MPa) [50], suggesting that, by using the P(AM-AA) as a superplasticizer, the compressive strength of brushite cements could meet the requirements for acrylic resin cements (ISO 5833) (Fig. S14C and D). The addition of P(AM-AA) showed no obvious influence on Young’s modulus of cements (Fig. 3G). The initial and final setting times decreased with the increase of the P(AM-AA) concentration (Fig. 3H to J).

Furthermore, superplasticizers suitable for apatite cements were screened (Fig. S15). The addition of AA/AMPS, P(AM-AA), PGA, and AA/HPA at a concentration of 5% (wt %) significantly enhanced the cement injectability, and these superplasticizers were selected for further evaluation. These superplasticizers markedly improved injectability irrespective of the L/P ratio (Figs. S16 and S17). Additionally, the incorporation of AA/AMPS, P(AM-AA), and AA/HPA slightly extended the setting time (Fig. S18), while the addition of PGA resulted in a slight reduction. The mechanical strength of the apatite cement was significantly enhanced with the addition of P(AM-AA), PGA, and AA/HPA when their concentrations exceeded 5 wt % (Fig. S19). Among these, the apatite cement modified with 5 wt % AA/AMPS exhibited the highest compressive strength and was further tested at an L/P ratio of 0.3 ml/g, leading to improved compressive strength, which increased with the hydration time (Fig. S20A). Phase composition analysis revealed a gradual reduction in the amount of raw material (α-TCP) and an increase in the apatite phase (Fig. S20B). These results highlight the universal applicability of the superplasticizer addition strategy in producing high-performance CPCs with improved injectability and mechanical strength.

### Water reduction mechanism

**Fig. 4.**
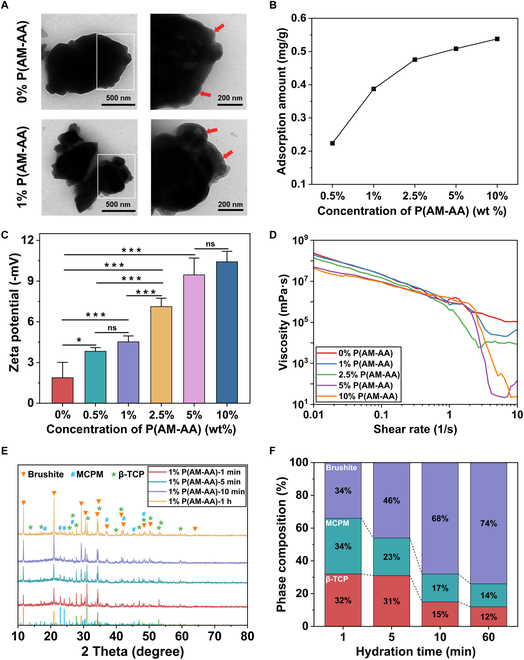
Investigation of the water reduction mechanism of the P(AM-AA) superplasticizer. (A) TEM images of brushite cements with or without P(AM-AA). (B) Adsorbed amount of P(AM-AA) on brushite cement particles at various superplasticizer concentrations. (C) Zeta potential of brushite cement particles at various superplasticizer concentrations. (D) Change in the viscosity of pastes with the shear rates at various P(AM-AA) concentrations. (E) Evolution of crystalline phases in the brushite cement with different hydration times. (F) Phase composition of brushite cements at different hydration intervals. The patterns are matched with the standard diffraction patterns of brushite (PDF #01-072-0713), MCPM (PDF #00-009-0347), and β-TCP (PDF #96-151-7239). The error bars represent the SD obtained by 3 independent repeated measurements. Statistical analysis was performed using one-way ANOVA, with significance defined as *P < 0.05, **P < 0.01, ***P < 0.001.

We further investigated the water reduction mechanism of P(AM-AA). The morphology of brushite cement hydrated for 24 h was analyzed using transmission electron microscopy (TEM), revealing that the thickness of the hydration shell around brushite cement particles increased with the addition of the P(AM-AA) superplasticizer (Fig. 4A). The total organic carbon (TOC) analysis indicated that the P(AM-AA) adsorption on brushite cement gradually increased, reaching saturation at a 10 wt % concentration (Fig. 4B). Zeta potential measurements of cement suspensions without P(AM-AA) exhibited negative values; however, as the P(AM-AA) concentration increased, the negative zeta potential on the cement particles also increased (Fig. 4C), reducing agglomeration via electrostatic repulsion, and effectively improving the dispersion and stability of the cement paste [20,51]. The viscosity of cement pastes changed with the P(AM-AA) concentration, exhibiting shear-thinning behavior (viscosity of 0% and 1% groups remains consistent within the range of 0.02 to 0.24; viscosity of 5% and 10% groups remains consistent within the range of 0.17 to 0.33), a characteristic of non-Newtonian fluids. As the shear rate increased, the liquid trapped within the cement particles was gradually released, leading to a higher free liquid content and reduced viscosity. Compared to pastes without superplasticizers, those containing P(AM-AA) exhibited significantly lower viscosity across all shear rates (Fig. 4D). This reduction is attributed to the stronger electrostatic repulsion at higher superplasticizer concentrations, which helps release water molecules from the aggregates, lowering both particle concentration and viscosity [52,53]. The hydration products at various hydration times were quantitatively analyzed by Rietveld refinement of powder x-ray diffraction (PXRD) patterns, showing that the amount of brushite progressively increased, reaching 74% after 60 min (Fig. 4E and F).

### In vitro performance of the cements

**Fig. 5.**
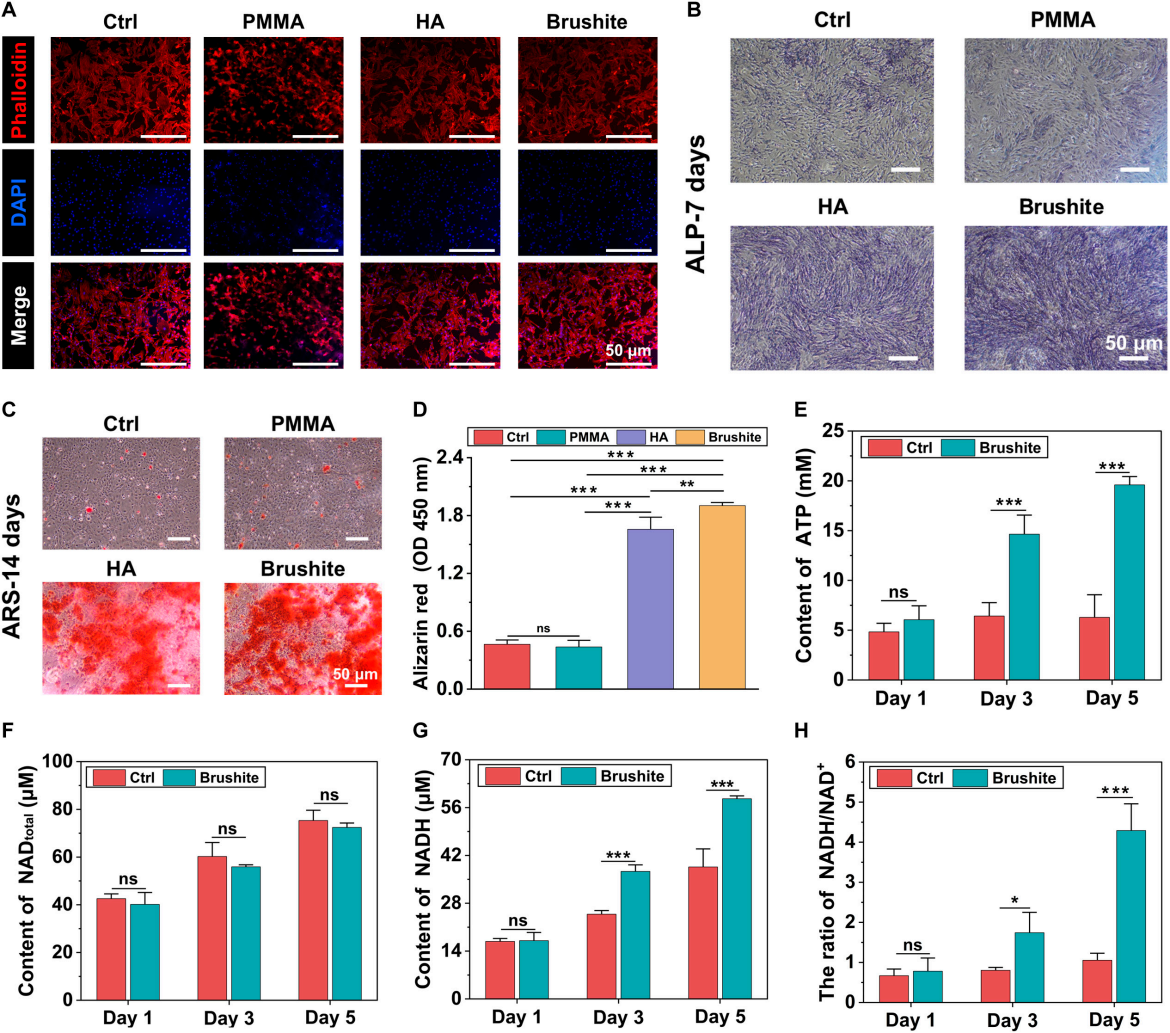
Osteogenic ability and citrate metabolic regulation in vitro. (A) Cytoskeletal staining of BMSCs exposed to extracts of different cements. (B) Alkaline phosphatase (ALP) staining and (C) Alizarin red staining (ARS) of BMSCs cultured with extracts of PMMA, HA, and brushite cements for 7 and 14 d, respectively. (D) Quantitative analysis of ARS. (E) Intracellular ATP levels of BMSCs cultured with the extract of brushite cements for different times compared to the Ctrl. (F) Changes in intracellular NAD_total_ content, (G) NADH content, and (H) NADH/NAD^+^ ratios of BMSCs cultured with the extract of brushite cement for 1, 3, and 5 d. “Ctrl” denotes the cells cultured without cement extracts. Error bars represent the SD from 3 independent measurements. Statistical analysis was performed using unpaired parametric Student’s *t* test and ordinary one-way ANOVA in OriginLab. *P < 0.05, **P < 0.01, ***P < 0.001.

High-performance brushite cements [liquid phase: 0.14 M AIC + 1% P(AM-AA)] was used for in vitro experiments. Therefore, the in vitro performance of brushite cements was evaluated alongside apatite cements [hydroxyapatite (HA), liquid phase: 2.5% NaH_2_PO_4_] and PMMA cements. Bone marrow stem cells (BMSCs) exhibited healthy morphology in the brushite and HA groups, while the cytoskeleton in the PMMA group appeared contracted (Fig. 5A and Fig. S21). Biocompatibility results indicated that PMMA, HA, and brushite cements were nontoxic to BMSCs (Fig. S22). Furthermore, BMSC proliferation was enhanced in both the HA and brushite groups during culturing (Fig. S23). The in vitro osteogenic property of brushite cements was evaluated using alkaline phosphatase (ALP) and Alizarin red staining (ARS). Both brushite and HA cements demonstrated significantly higher ALP expression and greater quantities of mineralized nodules than PMMA and Ctrl groups, with brushite exhibiting the highest levels (Fig. 5B and C). The addition of citrate may enhance the osteogenic effect of brushite cement. Quantitative analyses of ALP activity and ARS further validated that the osteogenic ability of brushite cements surpassed that of the other groups (Fig. 5D and Fig. S24). For polymer-reinforced apatite bone cement, most [polyvinyl alcohol (PVA) fibers and poly(lactic acid) (PLA)] have good biocompatibility and osteogenic properties [54,55]. However, some polymer-reinforced apatite bone cements exhibited obvious toxicity [56,57]. Therefore, the use of superplasticizer also needs to consider potential toxicity or adverse effects at high concentrations. For example, AA/AMPS and AA/HPA polymers have acidic pH levels, which can exert obvious toxicity on cells. Polymers such as PSA, SL, and TES are nontoxic at low concentrations, but they can also exert toxic effects on cells when the concentrations are high. In contrast, P(AM-AA) and PGA exhibit excellent biocompatibility and are widely used for the modification of biomaterials [58,59]. Furthermore, the high-performance brushite cement modified by 1% P(AM-AA) and AIC not only has good biocompatibility but also can promote osteogenic differentiation.

Citrates are commonly used as a setting retarder for brushite cement and are an essential component of bones, comprising 1 to 5 wt % of its organic matrix [44]. They play a critical role in bone development by regulating mineralization, energy metabolism, and various biological functions, including angiogenesis and osteogenic differentiation [43,60,61]. Additionally, as a vital substrate for energy metabolism, citrates enhance the generation rate and yield of ATP [62,63]. Nicotinamide adenine dinucleotide (NAD^+^) and its reduced form NADH, both hydride-donating coenzymes, also participate in energy metabolism [64]. To investigate the impact of brushite on the energy metabolism and anabolism of BMSCs, we measured intracellular ATP and NADH/NAD^+^ levels. The ATP levels in the Ctrl group remained stable at approximately 5 to 8 mM over 5 d, whereas ATP levels in the brushite group increased significantly, reaching 19.5 mM by day 5 (Fig. 5E). Moreover, the intracellular NADH content and the NADH/NAD^+^ ratio were markedly elevated after culturing with brushite cement extracts for 3 and 5 d (Fig. 5F to H). These results demonstrate that citrates can regulate the intracellular energy metabolism of BMSCs, increasing the energy level for their osteogenic differentiation.

### Potential signaling pathway for the therapeutic effects of brushite cements

**Fig. 6.**
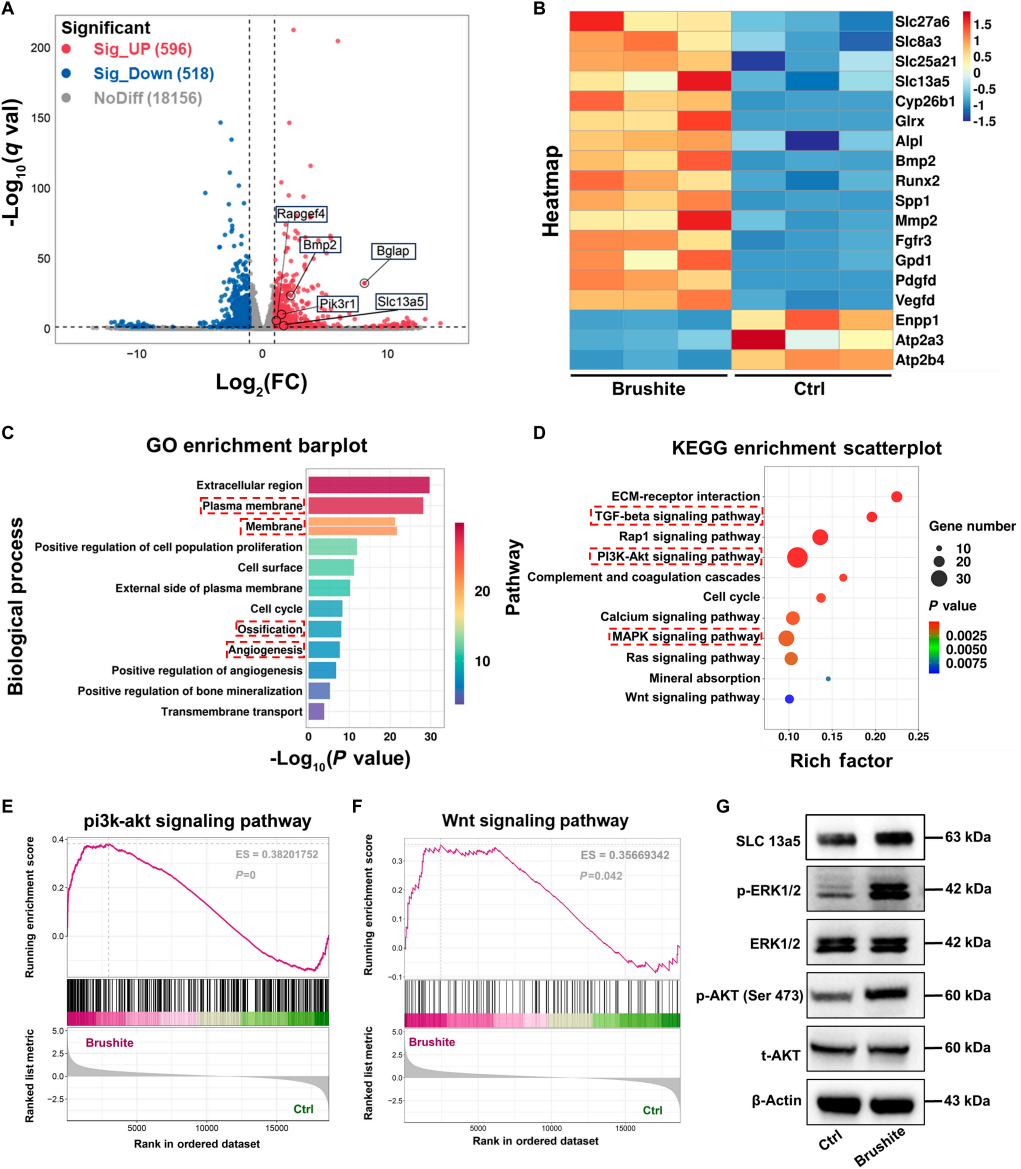
Transcriptomic analysis. (A) Volcano plot of differentially expressed genes (DEGs). Red dots indicate up-regulated genes; blue dots indicate down-regulated genes; gray dots indicate unchanged genes. (B) The heatmap shows a series of DEGs related to multicellular organism development, extracellular matrix organization, and structural constituent of ribosomes in BMSCs. (C) GO analysis shows the main biological processes involved in DEGs. (D) KEGG enrichment bubble plot shows the main pathways involved in DEGs. (E and F) GSEA shows that the “pi3k-akt signaling pathway” and “Wnt signaling pathway” were up-regulated in the brushite cement group. (G) Western blotting of t-AKT and p-AKT, ERK and p-ERK, and SLC13a5. “Ctrl” denotes the cells cultured without cement extracts.

RNA sequencing was performed to investigate the potential molecular mechanisms underlying the therapeutic and regenerative effects of brushite cement. A total of 596 differentially expressed genes (DEGs) were up-regulated, while 518 DEGs were down-regulated in the brushite group compared with the Ctrl group, as depicted in a volcano plot (|log2FC| ≥ 1 and *P* < 0.05) (Fig. 6A). From the heatmap results, the solute carrier (SLC)-related genes (e.g., *Slc27a6, Slc8a3, Slc25a21*, and *SLC13a5*) were obviously up-regulated in the brushite group. The increase in the citrate content transported by SLC accelerated energy metabolism and anabolism [65,66]. ATP degradation-related genes *Enpp1*, *Atp2a3*, and *Atp2b4* were down-regulated (Fig. 6B). The up-regulated expression of SLC can promote the transmembrane transport of citrate [46] and ATP degradation-related gene *Enpp1*, which can reduce the degradation of ATP, meeting the energy requirements for BMSC proliferation, osteogenesis, and bone mineralization [67,68]. The expression levels of many osteogenesis-related genes (e.g., *Bmp2*, *Alpl*, *Spp1*, and *Runx2*) were dramatically up-regulated in the brushite group. Gene ontology (GO) analysis verified that these DEGs were associated with transmembrane transport, ossification, positive regulation bone mineralization, and angiogenesis (Fig. 6C). Kyoto Encyclopedia of Genes and Genomes (KEGG) pathway enrichment analysis indicated that many signaling pathways associated with bone development were up-regulated in the brushite group, such as transforming growth factor-β (TGF-β), phosphatidylinositol 3-kinase (PI3K)–AKT, and mitogen-activated protein kinase (MAPK) signaling pathways (Fig. 6D). Furthermore, gene set enrichment analysis (GSEA) showed that the PI3K–Akt signaling pathway and Wnt signaling pathway were all markedly up-regulated in the brushite group (Fig. 6E and F). These signaling pathways were crucial for the osteogenic differentiation of BMSCs and for promoting bone mineralization [69,70]. Further studies by Yang and colleagues [44] found that citrate may modulate cytosolic acetyl-CoA (coenzyme A) levels, thereby influencing multiple critical intracellular activities during osteogenic differentiation, including histone acetylation and methylation, nonhistone protein acetylation, and directional lipid metabolism, synergistically promoting bone tissue regeneration. Furthermore, exogenous citrate supplementation can enhance energy metabolism in mononuclear macrophages to drive osteoclast differentiation, which subsequently secretes cytokines that stimulate osteoblast differentiation. Hence, the expression levels of SLC13a5, extracellular signal–regulated kinase (ERK), total AKT (t-AKT), phosphorylated ERK (p-ERK), and phosphorylated AKT (p-AKT) were verified using Western blotting. p-ERK, p-AKT, and SLC13a5 were up-regulated in the brushite group compared with the Ctrl group (Fig. 6G), indicating the activation of PI3K-AKT and MAPK-ERK signaling pathways and the enhancement of the energy metabolism. In summary, the brushite cement can encourage the osteogenic differentiation of BMSCs by boosting energy metabolism and stimulating PI3K-AKT and MAPK-ERK signaling pathways.

### In vivo study

**Fig. 7.**
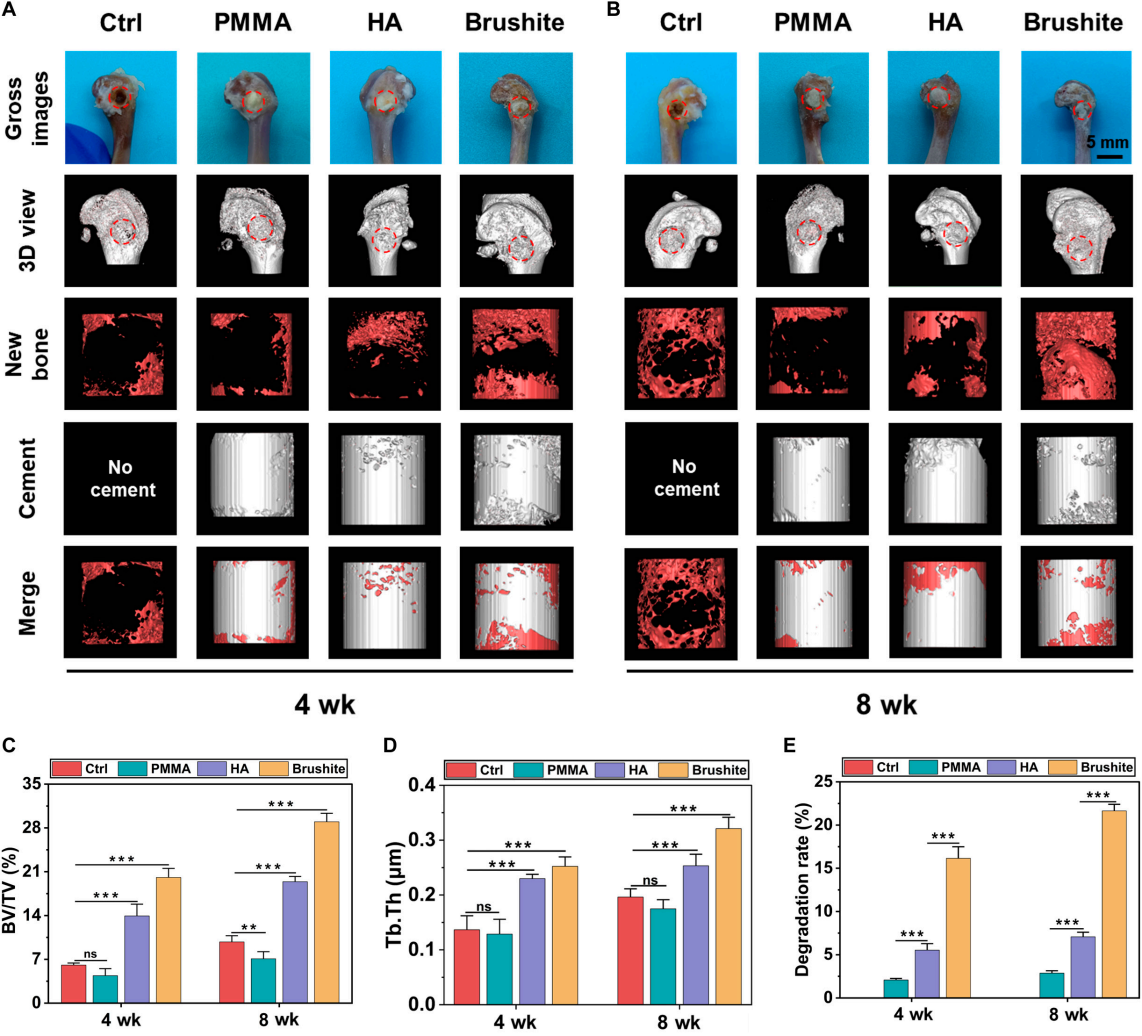
Bone repair effect of different cements in vivo. Gross images and micro-CT images of femoral defects after (A) 4 and (B) 8 weeks. (C) BV/TV value of femoral defect repair at 4 and 8 weeks. (D) Tb.Th value of femoral defect repair at 4 and 8 weeks. (E) Degradation rates of different cements in vivo. “Ctrl” denotes the repair femoral defect without cements. Error bars represent SDs obtained by 3 independent repeated measurements. Statistical analysis was performed using one-way ANOVA, with significance defined as *P < 0.05, **P < 0.01, ***P < 0.001.

The osseointegration and degradation of bone cement are crucial for repairing bone defects [71–73]. PMMA cement, as a nondegradable biomaterial, hinders new bone ingrowth [74,75]. In contrast, brushite cement offers superior degradability [27]. Our results align with previous studies, confirming that brushite cement promotes the highest bone ingrowth at both 4 and 8 weeks (Fig. 7A and B), reaching at these time intervals a degradation rate of 16 and 21%, respectively (Fig. 7E). The quantitative assessment of the bone volume/total volume (BV/TV) and trabecular thickness (Tb.Th) further illustrates the superior osteogenic properties of brushite cement compared to HA and PMMA cements (Fig. 7C and D).

**Fig. 8.**
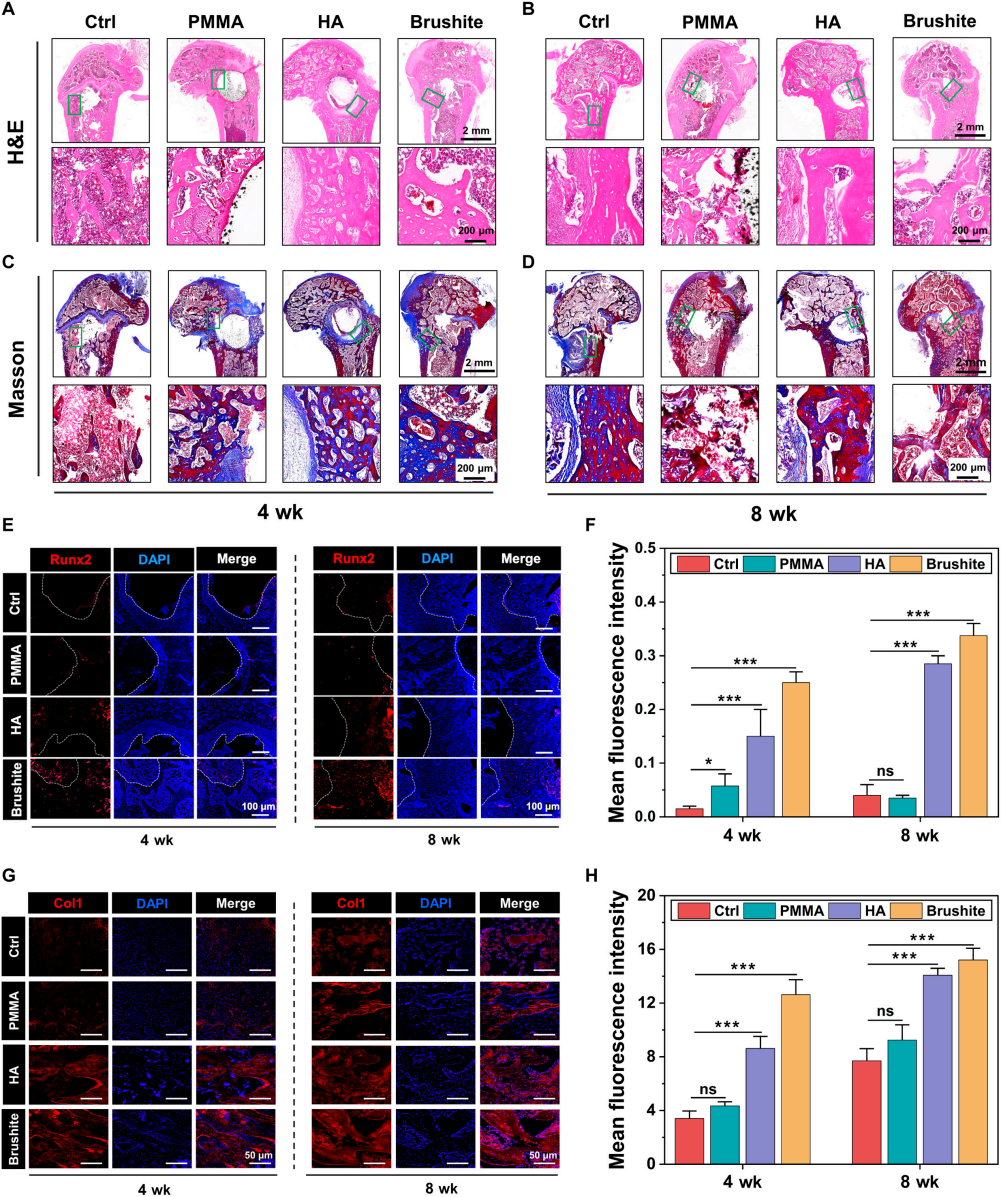
Histological staining of a femoral defect in vivo. The H&E staining of the femur sections after (A) 4 and (B) 8 weeks. Masson staining of the femur sections after 4 (C) and 8 weeks (D). (E) Immunofluorescent staining of Runx2 of a femoral section after 4 and 8 weeks. (F) Quantitative analysis of the mean fluorescent intensity of Runx2. (G) Immunofluorescent staining of Col1 of a femoral section after 4 and 8 weeks. (H) Quantitative analysis of the mean fluorescent intensity of Col1. “Ctrl” denotes the repair femoral defect without cements. Error bars represent the SD obtained by 3 independent repeated measurements. Statistical analysis was performed using ordinary one-way ANOVA tests with OriginLab. *P < 0.05, **P < 0.01, ***P < 0.001.

Histological staining was conducted on nondecalcified and decalcified samples to evaluate the osteogenic ability and osseointegration of the investigated cements. In the Ctrl group, the femoral defect was occupied with soft tissue and a small amount of new bone. HA cement was surrounded by a substantial amount of soft tissue composed of collagen and muscle fibers. In contrast, PMMA and brushite cement groups showed a close fit at the defect edges, with no soft tissue between the cements and bone. Notably, new bone formation was observed around the brushite cements (Fig. S25). Histological analysis of decalcified femurs revealed only a small amount of new bone in PMMA and HA cement groups at both 4 and 8 weeks (Fig. 8A and B), primarily due to the limited degradation of these cements in vivo. In contrast, the brushite cement group exhibited higher amounts and maturity of newly formed bone compared to the other groups (Fig. 8C and D).

Moreover, the IF staining of osteogenic protein [runt-related transcription factor 2 (Runx2) and collagen I (Col1)] was used to evaluate osteogenic ability. As important osteogenic proteins, Runx2 and Col1 play a crucial role in bone matrix mineralization. The results of IF show that HA and brushite cements significantly up-regulated the expression of Runx2 after 4 and 8 weeks (Fig. 8E and F). There was no difference between the Ctrl and PMMA cement, plausibly because the citrate released from brushite cement promoted the expression of Runx2. As for the expression of Col1, no obvious difference appeared between the Ctrl and PMMA cement, and the Col1 expression in HA and brushite cements increased after 4 and 8 weeks (Fig. 8G). The level of Col1 expression of HA cement was consistent with that of brushite cement after 8 weeks, both being higher than those of the Ctrl and PMMA cement (Fig. 8H).

## Materials and Methods

### Materials

β-TCP and α-TCP were purchased from Suzhou Dingan. MCPM was obtained from Scharlau. PMMA (OSTEOPAL V) was sourced from Heraeus. AIC and P(AM-AA) were acquired from Sigma-Aldrich. AA/HPA and SL were purchased from Macklin. PSA was obtained from Adamas. TES was sourced from J&K Scientific. AA/AMPS and P(AA-MA) were obtained from Titan Scientific. PGA was sourced from Saitaisi Biotechnology.

### Data collection

To construct ML models for analyzing the data statistically and predicting outcomes, we collected data from our laboratory experiments. Data were collected from 1,440 (8 × 4 × 5 × 3 × 3) experimental samples, which contained 8 different polymers with different values of concentration, compressive strength, initial setting time, final setting time, and injectability. The initial setting time and final setting time were measured for the same arbitrary sample. The setting time, injectability, and compressive strength were measured for 3, 3, and 5 times, respectively.

### ML models and evaluation

Before model training and predication, the collected data were subject to an evaluating step in which measurements that appeared to be corrupted by obvious operational errors/sample defects were removed. Four different ML models were utilized to screen superplasticizers, namely, RF, DT, SVM, and FCNN.

#### Random forest

RF is an ensemble method based on DTs, which trains multiple trees through bagging and votes/averages the results. Key hyperparameters include n_estimators, random_state, max_depth, and max_features, where n_estimators is set to 100, random_state = 42, and max_depth and max_features are left as defaults. Feature selection relies on the feature_importances algorithm, and cross-validation is employed for evaluation.

#### Decision tree

DT is a tree-structured model that recursively splits data for classification/regression. Hyperparameters such as max_depth and min_samples_split need tuning to prevent overfitting, with max_depth set to 3 and min_samples_split to 2. Feature selection uses feature importance analysis and recursive feature elimination (RFE), while validation relies on *K*-fold cross-validation and confusion matrices.

#### Fully connected neural network

FCNN consists of a classification network and a regression network, both containing input, hidden, and output layers. The classification part has 10 neurons in the hidden layer and 8 in the output layer, while the regression part has 10 hidden neurons and 4 output neurons. The loss function combines CrossEntropyLoss and MSELoss(), and the Adam optimizer is used with a learning rate of 0.00001. Features are extracted from intermediate hidden layers, and cross-validation is applied for model evaluation.

#### Support vector machine

SVM includes both support vector regression (SVR) and support vector classification (SVC). SVR predicts continuous variables (e.g., initial setting time, final setting time, injectability, and compressive strength), while SVC predicts discrete variables (grouping). Nonlinear relationships are handled using the Gaussian/radial basis function (RBF) kernel, and cross-validation is used for model assessment.

These models were operating using a Python programming environment. The collected data were divided into training data and testing data, each accounting for 80% and 20%, respectively. Coefficient of determination (*R*^2^), root mean square error (RMSE), and MSE were employed to evaluate the performance of models according to Eqs. 1 to 3:

$$R^{2}=1-\frac{\sum_{p=1}^{m}{\left(Z_{p}-z_{p}\right)}^{2}}{\sum_{p=1}^{m}{\left(z_{p}-\bar{z}\right)}^{2}}$$ (1)

$$\text{RMSE}=\frac{1}{m}\sum_{p=1}^{m}\sqrt{{\left(Z_{p}-z_{p}\right)}^{2}}$$ (2)

$$\mathrm{MSE}=\frac{1}{m}\sum_{p=1}^{m}{\left(Z_{p}-z_{p}\right)}^{2}\;$$ (3)

where *m* is the total sample count, $\bar{z}$ is the arithmetic mean of the experimental values, 𝑧 is the actual experimental value, and *Z* is the regression-based predicted value.

### Preparation of cements

#### Brushite cement

The cement powder phases consisted of β-TCP and MCPM in a 1:1 ratio, while the liquid phase comprised 0.26 M AIC and P(AM-AA) at varying concentrations. The cement was prepared by sieving MCPM to obtain particles with a diameter of less than 75 μm. First, β-TCP and MCPM were evenly mixed for 20 min using an oscillator. Next, the liquid phase (L) was combined with the powder phase (P) at specific L/P ratios (0.3, 0.25, and 0.225). The resultant paste was then filled into polydimethylsiloxane (PDMS) molds and allowed to set at 37 °C for 2 h. Finally, the cements were immersed in PBS at 37 °C and left overnight.

#### HA cement

The powder phase was α-TCP, and it was uniformly mixed with a liquid phase of 2.5% (w/v) NaH₂PO₄ at different L/P ratios. The resulting mixture was then filled into PDMS molds.

#### PMMA cement

The powder phase consisted of zirconium dioxide, benzoyl peroxide, and poly (methyl acrylate, methyl methacrylate). The liquid phase was composed of *N*,*N*-dimethyl-*p*-toluidine and methyl methacrylate. PMMA cement was fabricated by uniformly mixing the liquid and powder phases at certain L/P ratios.

The preparation of cements modified by superplasticizers involved creating brushite cements (liquid phase: 0.55 M AIC, powder phase: β-TCP and MCPM) and apatite cements (liquid phase: 2.5 wt % NaH₂PO₄, powder phase: α-TCP) in specified proportions. The powder and liquid phases, each containing varying concentrations of superplasticizers, were then mixed at different L/P ratios.

### Characterizations of cements

#### Setting time

The setting time was determined by the Gillmore needle. Powder and liquid phases were thoroughly blended and filled into stainless steel rings (5 mm in height, 10 mm in diameter). The needle was applied to the cement surface for 5 s, and the setting time was recorded when no mark was left by the needle. Initial and final setting times were measured using the light needle (2.12 mm/113.4 g) and the heavy needle (1.06 mm/453.6 g), respectively.

#### Compressive strength measurement

Powder and liquid phases of cements were thoroughly mixed and molded in PDMS molds with a diameter of 6 mm and a height of 12 mm. After setting at room temperature for 2 h, both cements and PDMS molds were immersed in PBS at 37 °C for predetermined times. The cement specimens were then removed from the PDMS molds and polished using 2000 grit sandpaper. A universal testing machine was applied to test the compressive strength at a speed of 1 mm/min. Each group was tested with a total of 6 samples.

#### Injectability test

The injectability of cements was evaluated using a universal testing machine. A 2.5-ml syringe was weighed empty (W0), and it was then filled with the mixed cement before being weighing again (Wt). The cement was pressed at a speed of 5 mm/min until the pressure reached 100 N, at which point the test was terminated. The syringe was weighed again (Wr), and the injectability was calculated using Eq. 4:

$$\text{Injectability}\;\left(\%\right)=\frac{Wt-Wr}{\;Wt-W0}\times 100\%$$ (4)

#### Scanning electron microscopy

Cement specimens were initially dried in a vacuum oven and then finely ground to a uniform powder. To enhance conductivity, the specimens were sputter-coated with a thin layer of gold at a current of 20 mA for 60 s. The morphology of cements was performed using a field-emission scanning electron microscope (S-400, Hitachi, Japan) at an acceleration voltage of 15 kV.

#### X-ray powder diffraction

An x-ray powder diffractometer (D8 Advance, Germany), equipped with a Cu Kα radiation source, was utilized to analyze the degree of crystallinity and the composition of cements. A cement powder was placed in a quartz sample holder and measured at 40 mA and 40 kV. X-ray powder diffraction (XRD) data were acquired over a 2θ range of 10° to 80°, and the results were analyzed using HighScore Plus software. Rietveld refinement was applied to analyze the phase compositions of prepared specimens with BGMN software (BGMN, Germany). The standard structures used for the refinement were PDF #00-003-0681 (α-TCP), PDF #96-151-7239 (β-TCP), PDF #00-009-0347 (MCPM), PDF #01-072-0713 (brushite), and PDF #01-086-1199 (hydroxyapatite).

#### Transmission electron microscopy

To investigate the early hydration process of cements, the samples were quenched in liquid nitrogen after setting for the determined time to halt the reaction. The cement powder was dispersed in ethyl alcohol and subjected to ultrasonic treatment for 5 min. The resulting suspension was then placed onto a copper grid and air-dried. TEM micrographs were captured using a JEM 2100F TEM (JEOL, Japan) at an acceleration voltage of 200 kV.

#### TOC content

The adsorption of P(AM-AA) was quantified using a TOC analyzer (TOC-L, Japan). Briefly, 1 g of cement sample was thoroughly mixed with 10 ml of P(AM-AA) solution at varying concentrations (0.5, 1, 2.5, 5, and 10%). The cement paste was then centrifuged at 8,000 rpm for 5 min. The supernatant was immediately filtered through a 0.22-μm filter and diluted with deionized water for TOC analysis.

#### Zeta potential

One gram of powder (β-TCP and MCPM in a 1:1 ratio) was added to 50 ml of P(AM-AA) solution at varying concentrations (0, 0.5, 1, 2.5, 5, and 10%) and stirred at 2,000 rpm for 5 min. The suspension was then allowed to stand for 2 h. Finally, the supernatant was collected for zeta potential testing using a Malvern Zetasizer Nano ZS90 (UK).

#### Rheological properties

Rheological properties of cements were evaluated using an Anton Paar rheometer (MCR90, Austria) at room temperature. A Peltier plate geometry (25 mm plate diameter, 2 mm gap) was used to prepare samples with a diameter of 20 mm. The viscosity of specimens was measured through steady shear tests, with shear rates ranging from 0.01 to 10 s^−1^.

#### Cell culture

Bone marrow mesenchymal stem cells (BMSCs) were extracted from rat femurs and cultured to passage 2 (P2) for subsequent experiments. BMSCs were cultured with extracts prepared from cement samples and α-MEM (minimum essential medium) at a ratio of 0.2 g/ml. The cement extracts were then filtered and sterilized using 0.22-μm sterile syringe filters. Finally, complete α-MEM was prepared by combining the cement extracts with 10% fetal bovine serum (Gibco, USA) and 1% penicillin–streptomycin (Gibco, USA). An osteogenic induction medium was formulated using complete α-MEM supplemented with 100 mM β-glycerophosphate, 1 μM dexamethasone, and 2 mM ascorbic acid.

### Cytoskeleton staining

BMSCs were cultured at a density of 1 × 10^4^ cells in a 24-well plate and cultured with cement extracts for 3 d. At the predetermined time, the medium was removed, and the cells were fixed using 4% paraformaldehyde (PFA). The cells were then incubated with a solution of 0.3% Triton X-100 and 3% bovine serum albumin for 60 min at 37 °C. Finally, F-actin and cell nuclei were stained using tetramethyl rhodamine isothiocyanate–phalloidin and 4′,6-diamidino-2-phenylindole (DAPI) (Thermo Fisher, USA). Images were acquired using an inverted fluorescence microscope (Zeiss, Germany).

### Biocompatibility assessment

Cell Counting Kit 8 (CCK-8) and Live-Dead staining were employed to assess the biocompatibility of cements. BMSCs were cultured in a 96-well plate with cement extracts at a density of 1 × 10^3^ cells. After 1, 3, and 5 d of culturing, the cement extracts were replaced with CCK-8 (Abcam, USA) and incubated for 120 min. Optical density (OD) values were then determined by a microplate reader (BioTek, USA) at 450 nm. After 3 d of culturing, calcein-AM/propidium iodide was applied for live-dead staining, and fluorescent images were captured using an inverted fluorescence microscope.

### In vitro osteogenic experiment

#### ALP staining

BMSCs were cultured in a 24-well plate and cultured with an osteogenic induction medium. The cells were maintained for 7 d, and the osteogenic induction medium was changed every 2 d. At the predetermined time, ALP was stained using a bromochloroindolyl phosphate-nitro blue tetrazolium ALP color development kit (Beyotime, China) for 5 to 30 min. The images of ALP staining were acquired using an optical microscope. The quantitative analysis of ALP activity was performed by an ALP assay kit.

#### ARS staining

ARS was used to visualize calcium nodules. Firstly, BMSCs were cultured in a 24-well plate for 2 weeks in an osteogenic induction medium. After 2 weeks, the cells were fixed using 4% PFA and stained with an Alizarin Red S Staining Kit (Beyotime, China). The images of ARS were captured using an optical microscope. Subsequently, Alizarin Red S was dissolved in 1% perchloric acid, and the absorbance was determined by a microplate reader at 542 nm.

### Measurement of energy metabolism

BMSCs were cultured at a density of 5 × 10^5^ cells in a 6-well plate and cultured with cement extracts for 1, 3, and 5 d. At the predetermined time points, the medium was removed, and 100 μl of lysis buffer was added to each well to lyse the BMSCs. The lysis buffer was repeatedly pipetted to ensure thorough cell lysis, followed by centrifugation at 12,000 rpm for 5 min. Then, the standard curve was constructed using NAD^+^/NADH and ATP standard solutions in an ice bath. Subsequently, the NAD^+^/NADH and ATP assay working solutions were prepared using manufacturer-provided reagents. The concentrations of NAD^+^/NADH and ATP were measured from the supernatant using assay kits (Beyotime, China).

### RNA sequencing

BMSCs cultured with brushite cement extracts were designated as the brushite group (experimental group), while pure BMSCs served as the Ctrl group. Both groups were cultured in an osteogenic induction medium. TRIzol reagent (Vazyme, China) was used to extract total RNA. RNA purification, library preparation, and sequencing were conducted by Hangzhou LC-Bio Technology. HISAT2 software was employed to map reads to the reference genome of Rattus. The mRNA expression levels were ascertained by employing StringTie software, which computes the FPKM (fragments per kilobase of exon model per million mapped fragments). Genes with a fold change (FC) of >2 and a *P* value of <0.05 were considered DEGs. GO analysis and KEGG analysis were displayed using DAVID software (https://david.ncifcrf.gov/). GSEA was conducted using OmicStudio tools (https://www.omicstudio.cn). Plots were generated using R version 4.1.3 (2022-03-10) on the OmicStudio platform (https://www.omicstudio.cn).

### Western blot analysis

BMSCs were cultured with extracts [α-MEM (Ctrl group), PMMA, HA, and brushite] for 7 d. The total proteins from BMSCs were extracted with a radioimmunoprecipitation assay lysis buffer (Solarbio, China). The protein concentration in each group was determined using a bicinchoninic acid (BCA) protein assay kit (Yeasen, China). An sodium dodecyl sulfate–polyacrylamide gel electrophoresis (SDS-PAGE) loading buffer (Epizyme, China) was used to mix with 15 μg of protein lysate of each sample, denatured at 100 °C for 5 min, separated on 10% SDS-PAGE gels, and then electroblotted onto nitrocellulose (NC) membranes (Beyotime, China). The 5% nonfat milk was used to block the samples for 2 h. The primary antibodies (diluted 1:1,000) were used to incubate these membranes at 4 °C overnight and then treated with horseradish peroxidase-conjugated secondary antibodies (Beyotime, China) at room temperature for 60 min. The protein bands were performed by a chemiluminescence imaging system (SHST, China). Primary antibodies were SLC13a5 (Santa Cruz Biotechnology, USA), ERK (Cell Signaling Technology, USA), t-AKT (Abcam, UK), p-ERK (Cell Signaling Technology, USA), p-AKT (Cell Signaling Technology, USA), and β-actin (Servicebio, China).

### Animal experiments

Animal experiments were approved by the Institutional Animal Care and Use Committee of Soochow University (SUDA20220913A03). The subjects were male Sprague–Dawley rats, with a total of 40 animals, 8 weeks old, weighing approximately 350 g. The rats were randomly categorized into 4 groups: Ctrl group (Ctrl), PMMA group (PMMA), apatite group (HA), and brushite group (Brushite). To better simulate the clinical application of bone cement in weight-bearing areas, the femoral condyle defect model was constructed. Initially, the rats were anesthetized with pentobarbital (15 to 40 mg/kg) via intraperitoneal injection. The skin was disinfected with iodophor and incised to expose the femur. A micro-bone drill was used to construct cylindrical bone defect (3 mm diameter, 3 mm height). The powder and liquid phases of cements were then uniformly mixed, and the mixed cements were filled into 2.5 ml syringe. Then, the brushite cements were injected into the bone defects by the 2.5-ml syringe. Finally, the surgical incision was closed and sterilized again. At 4 weeks, half of the rats from each group were randomly selected for femur collection. The remaining rats from all groups were sacrificed for femur collection at 8 weeks.

### Micro-CT analysis

After 4 and 8 weeks, Sprague–Dawley rats were euthanized using an overdose of pentobarbital. The femurs were harvested and fixed in 10% neutral buffered formalin for 48 h at 37 °C. The femurs were then scanned using micro-CT (Belgium, Germany) with parameters set to 65 kV, 385 μA, and a 1-mm Al filter. The obtained micro-CT images were analyzed using NRecon, Dataview, and CTAn software. Finally, the micro-CT images were 3D-reconstructed using Mimics software.

### Hard tissue section

Excess fat and muscle tissue were removed from the harvested femurs. The femurs were dehydrated by a gradient of ethanol (70%, 80%, 90%, 95%, 100%, 100%; each gradient for 3 d) and then immersed in xylene for 24 h. The femurs were subsequently embedded in resin, and the embedded samples were sectioned into slices approximately 150 μm thick using a hard tissue microtome (Leica, SP1600, Germany). Finally, the samples were polished to a thickness of 20 μm using a grinder (Leica, EXAKT 400CS, Germany) and stained with hematoxylin and eosin (H&E) (Sinopharm, China) and Goldner staining (Sinopharm, China).

### Histological and IF staining

Harvested femurs were decalcified using an ethylenediamine tetraacetic acid·2Na (EDTA·2Na) solution for 30 d, and the solution was changed every 3 d. After decalcification, excess tissue from the bones and surrounding viscera was trimmed, and the samples were dehydrated using a gradient of ethanol (Sinopharm, China) before being embedded in paraffin (Leica, Germany). The embedded samples were sectioned to a thickness of 7 μm using a microtome (Leica, Germany). The paraffin sections were dewaxed with xylene and rehydrated using 75% ethanol. Finally, H&E (Solarbio, China) and Masson’s trichrome staining (Solarbio, China) were performed according to the manufacturer’s instructions.

The paraffin sections of the samples were dewaxed and rehydrated. The sections were then incubated with trypsin solution for antigen retrieval and blocked with an immunostaining blocking solution for 1 h at 37 °C. Primary antibodies against Runx2 (1:500, Abcam, UK) and Col1 (1:1,000, Abcam, UK) were used to detect the expression of osteogenic proteins. After 2 h of incubation, a fluorescent secondary antibody (1:500, Abcam, UK) was applied to bind the primary antibodies, and DAPI was applied to stain cell nuclei. Finally, IF images were acquired using a positive fluorescence microscope (Zeiss, Germany).

### Statistical analysis

OriginPro 2024b (Northampton, USA) was used for all statistical analyses. Results were demonstrated as the mean ± SD. Differences were evaluated using unpaired parametric Student’s *t* test and one-way analysis of variance (ANOVA), followed by Tukey’s multiple comparisons test. Different statistical significance was defined as *P < 0.05, **P < 0.01, and ***P < 0.001.

## Data Availability

All data are available in the main text or the Supplementary Materials.
