# Supplementary material for: Machine Learning-Assisted Development of Injectable, Mechanically Robust, and Energy Metabolism-Modulating Brushite Cements
Source: Research (Wash D C). 2025 Jul 10;8:0776. doi: 10.34133/research.0776 (PMC12241799; doi:10.34133/research.0776)
Supplement: Supplementary 1 — Figs. S1 to S25 [file research.0776.f1.docx]

Supplementary Material

**Machine learning-assisted development of injectable, mechanically robust and energy metabolism-modulating brushite cements**

**
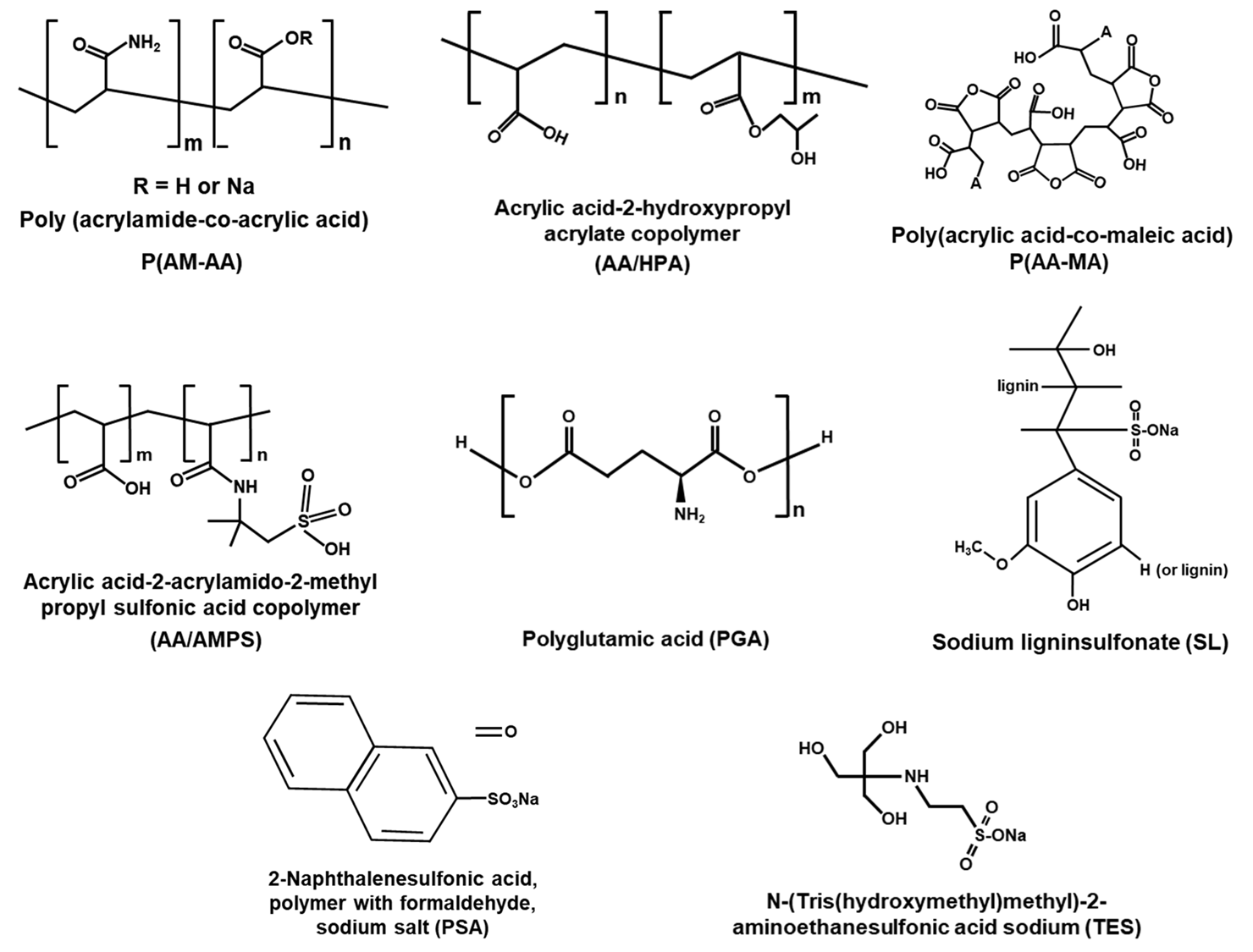
**

Fig. S1. Chemical structures of eight potential superplasticizers.


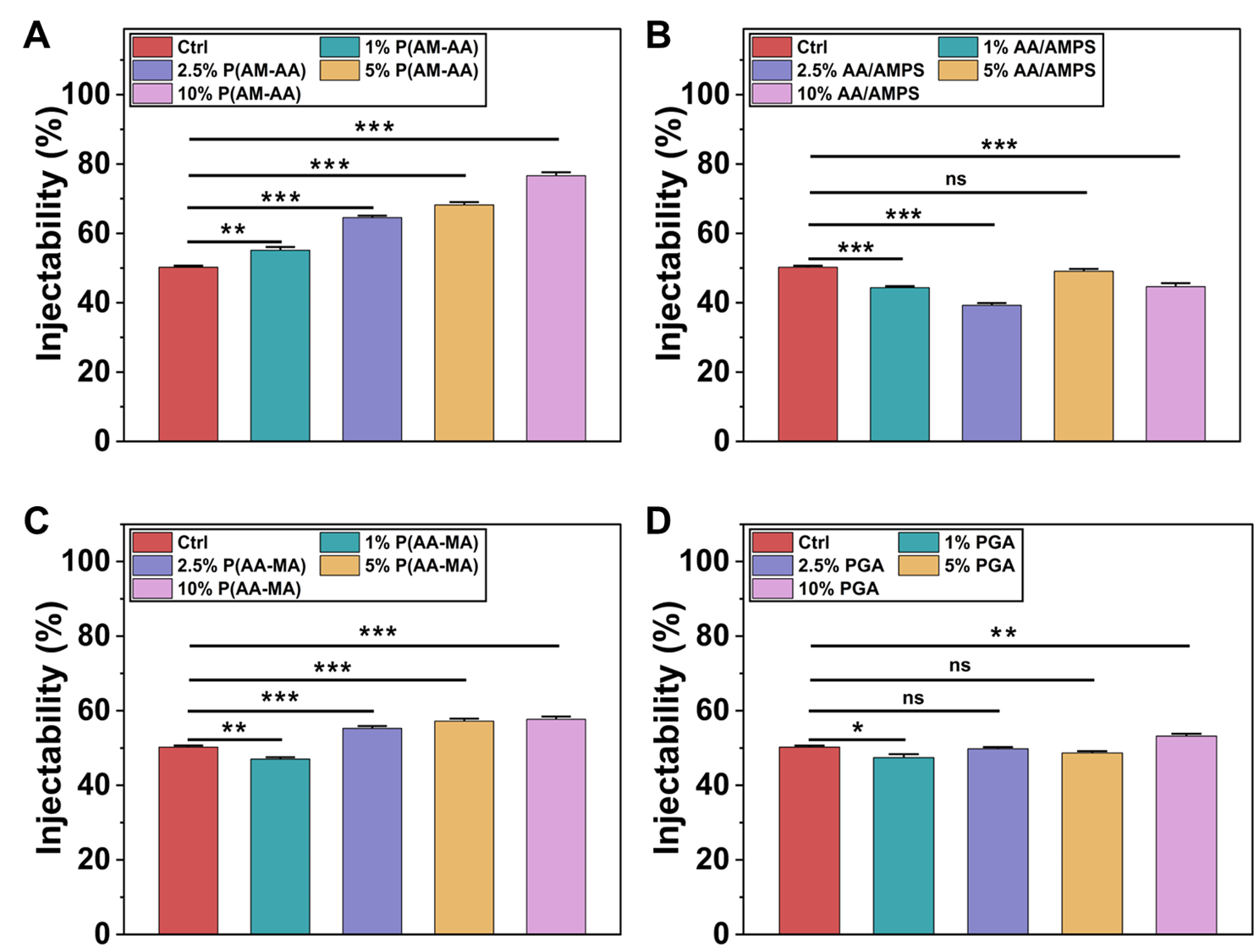


Fig. S2. Injectability of brushite cements (liquid phase: 0.55 M AIC + different wt.% amounts of superplasticizer; powder phase: β-TCP + MCPM) modified with different superplasticizers at an L/P of 0.25 mL/g. (A) P(AM-AA), (B) AA/AMPS, (C) P(AA-MA), and (D) PGA. Error bars represent standard deviations obtained by three independent repeated measurements (Ctrl: 0.55 M AIC).


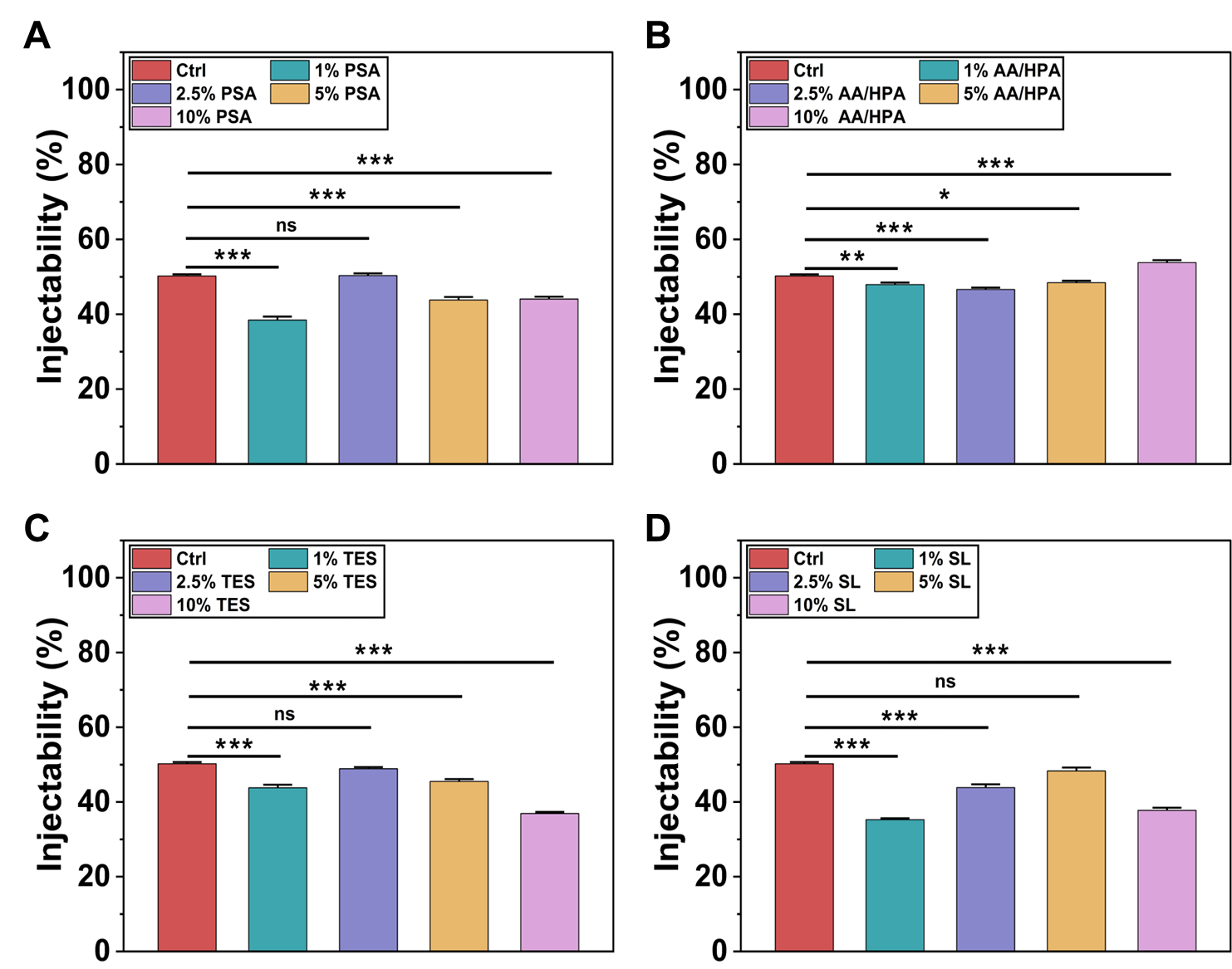


Fig. S3. Injectability of brushite cements (liquid phase: 0.55 M AIC + different wt.% amounts of superplasticizer, powder phase: β-TCP + MCPM) modified with different superplasticizers at an L/P of 0.25 mL/g. (A) PSA, (B) AA/HPA, (C) TES, and (D) SL. Error bars represent standard deviations obtained by three independent repeated measurements (Ctrl: 0.55 M AIC).

**
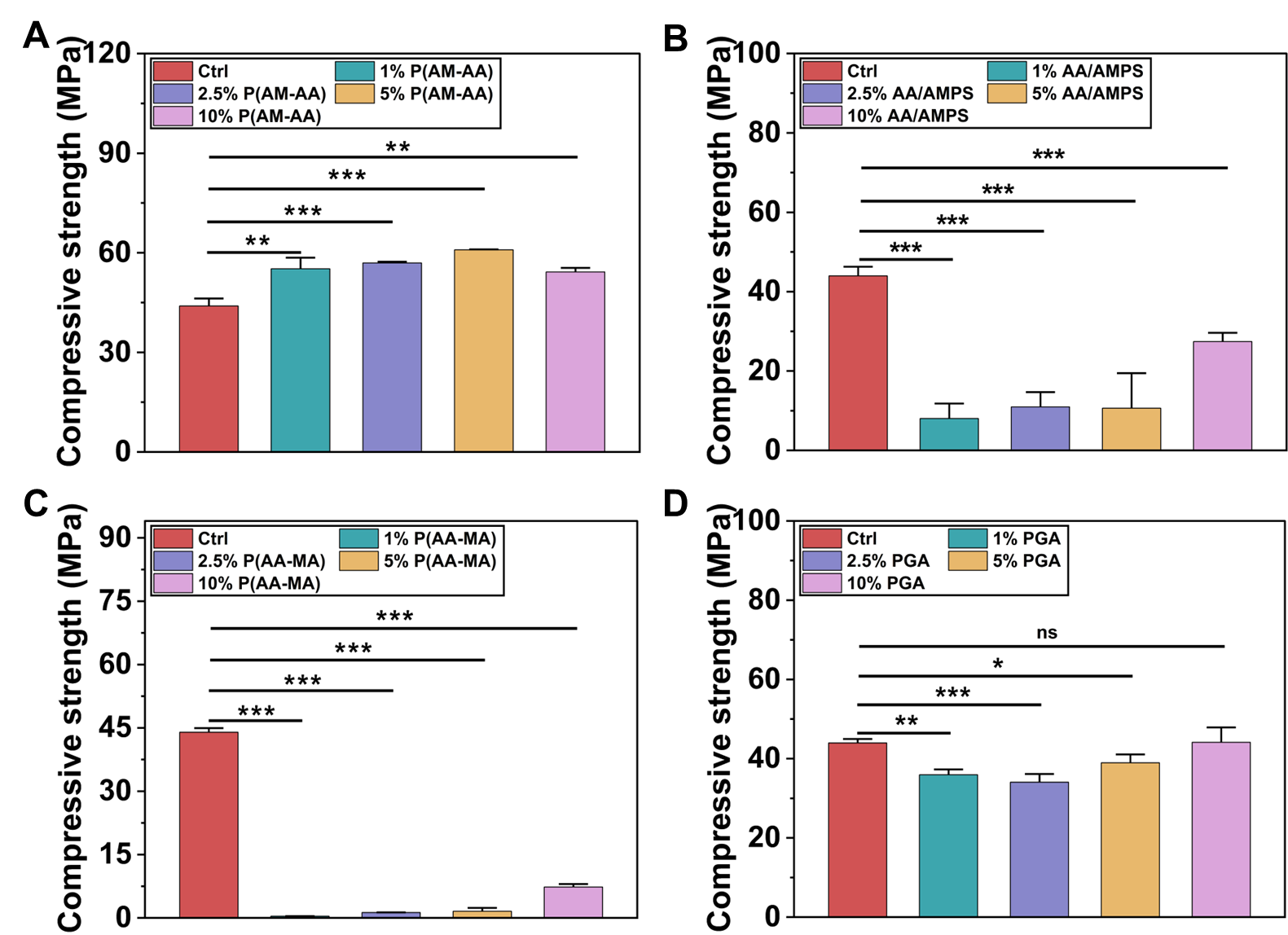
**

Fig. S4. Compressive strength of brushite cements (liquid phase: 0.55 M AIC + different wt.% amounts of superplasticizer, powder phase: β-TCP + MCPM). modified with different superplasticizers at an L/P of 0.25 mL/g. (A) P(AM-AA), (B) AA/AMPS, (C) P(AA-MA), and (D) PGA. Error bars represent standard deviations obtained by five independent repeated measurements (Ctrl: 0.55 M AIC).

**
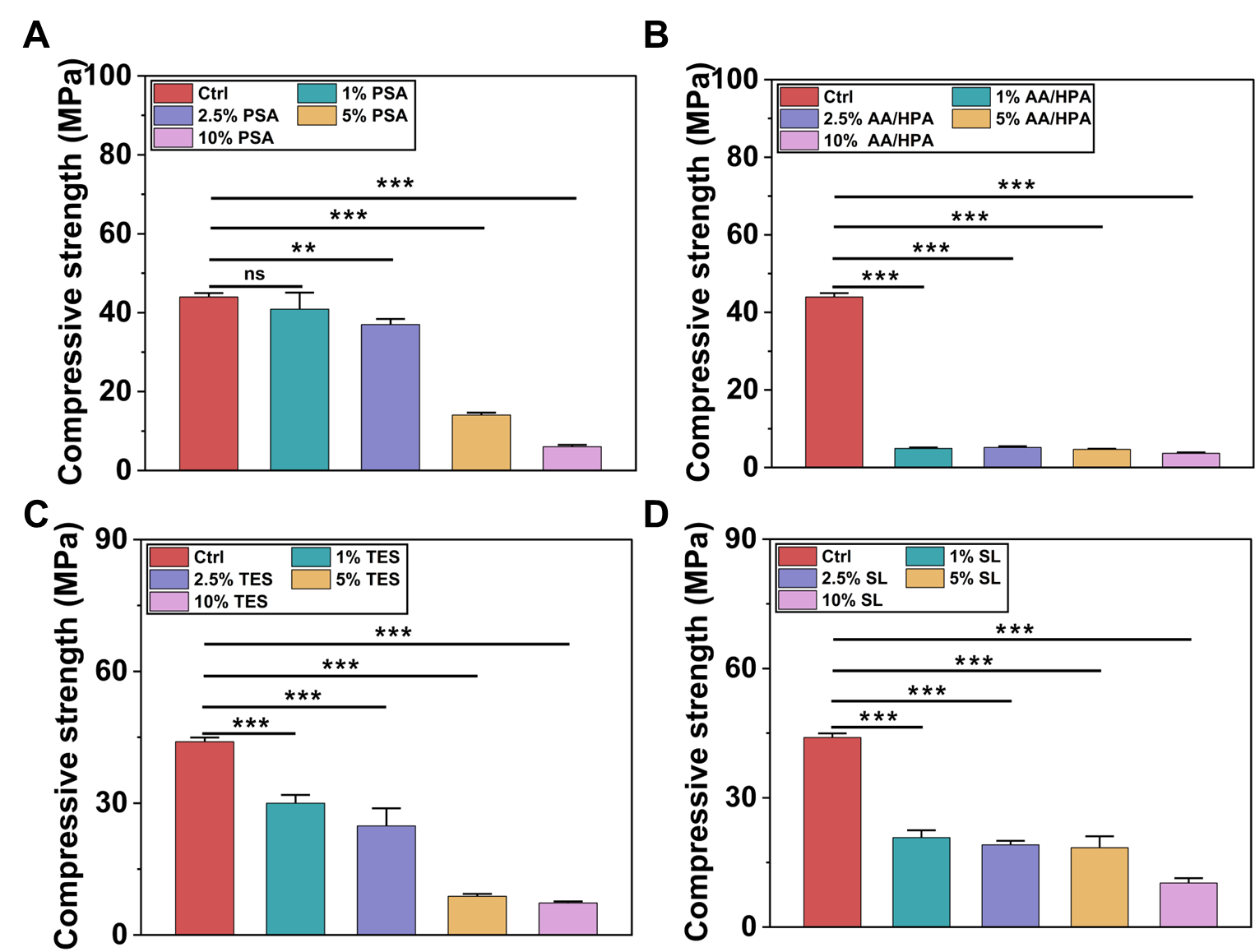
**

**Fig. S5.** Compressive strength of brushite cements (liquid phase: 0.55 M AIC + different wt.% amounts of superplasticizer, powder phase: β-TCP + MCPM) modified with different superplasticizers at an L/P of 0.25 mL/g. (A) PSA, (B) AA/HPA, (C) TES, and (D) SL. Error bars represent standard deviation obtained by five independent repeated measurements (Ctrl: 0.55 M AIC).

**
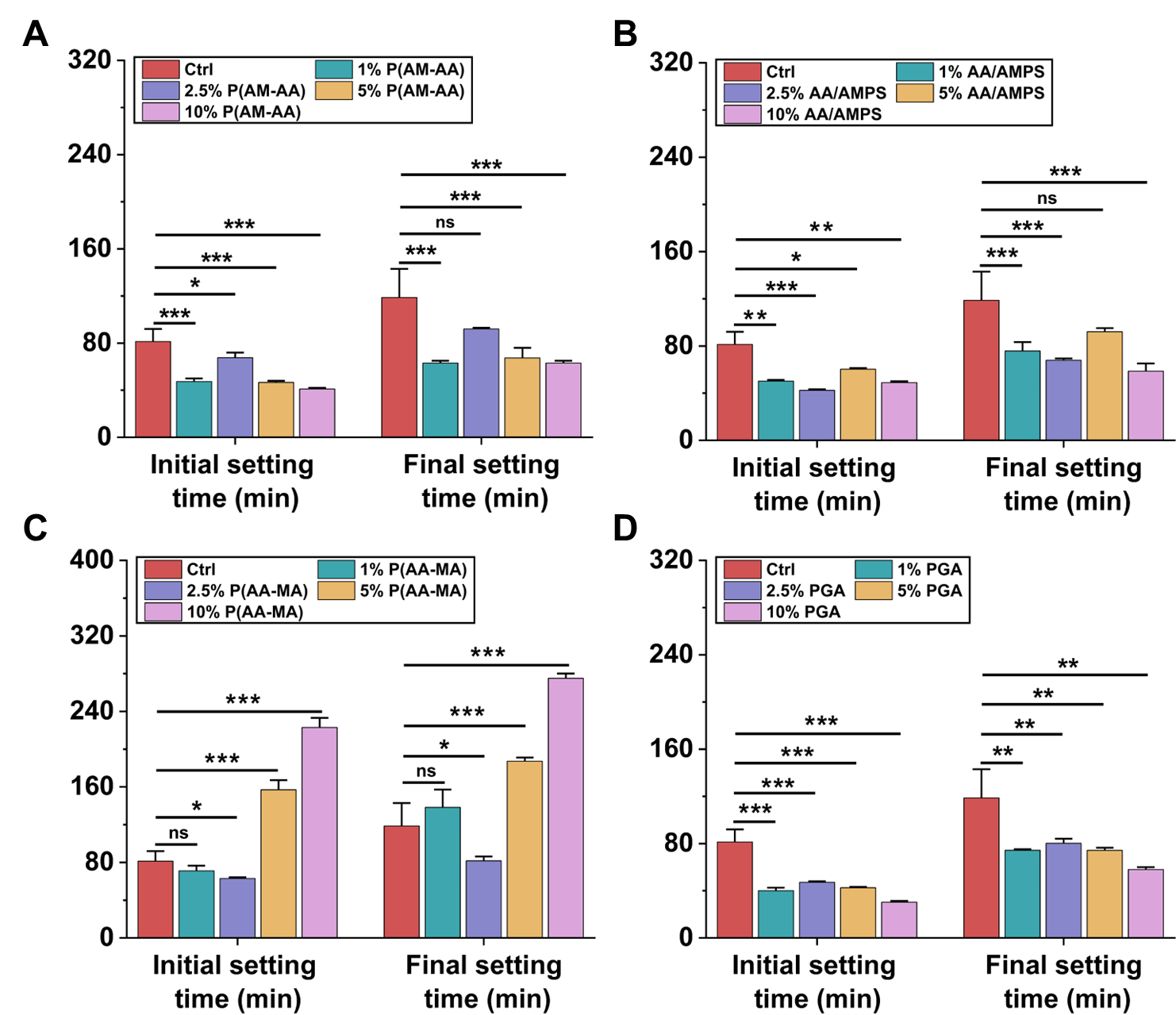
**

**Fig. S6.** Initial and final setting times of brushite cements (liquid phase: 0.55 M AIC + different wt.% amounts of superplasticizer, powder phase: β-TCP + MCPM). modified with different superplasticizers at an L/P of 0.25 mL/g. (A) P(AM-AA), (B) AA/AMPS, (C) P(AA-MA), and (D) PGA. Error bars represent standard deviations obtained by three independent repeated measurements (Ctrl: 0.55 M AIC).

**
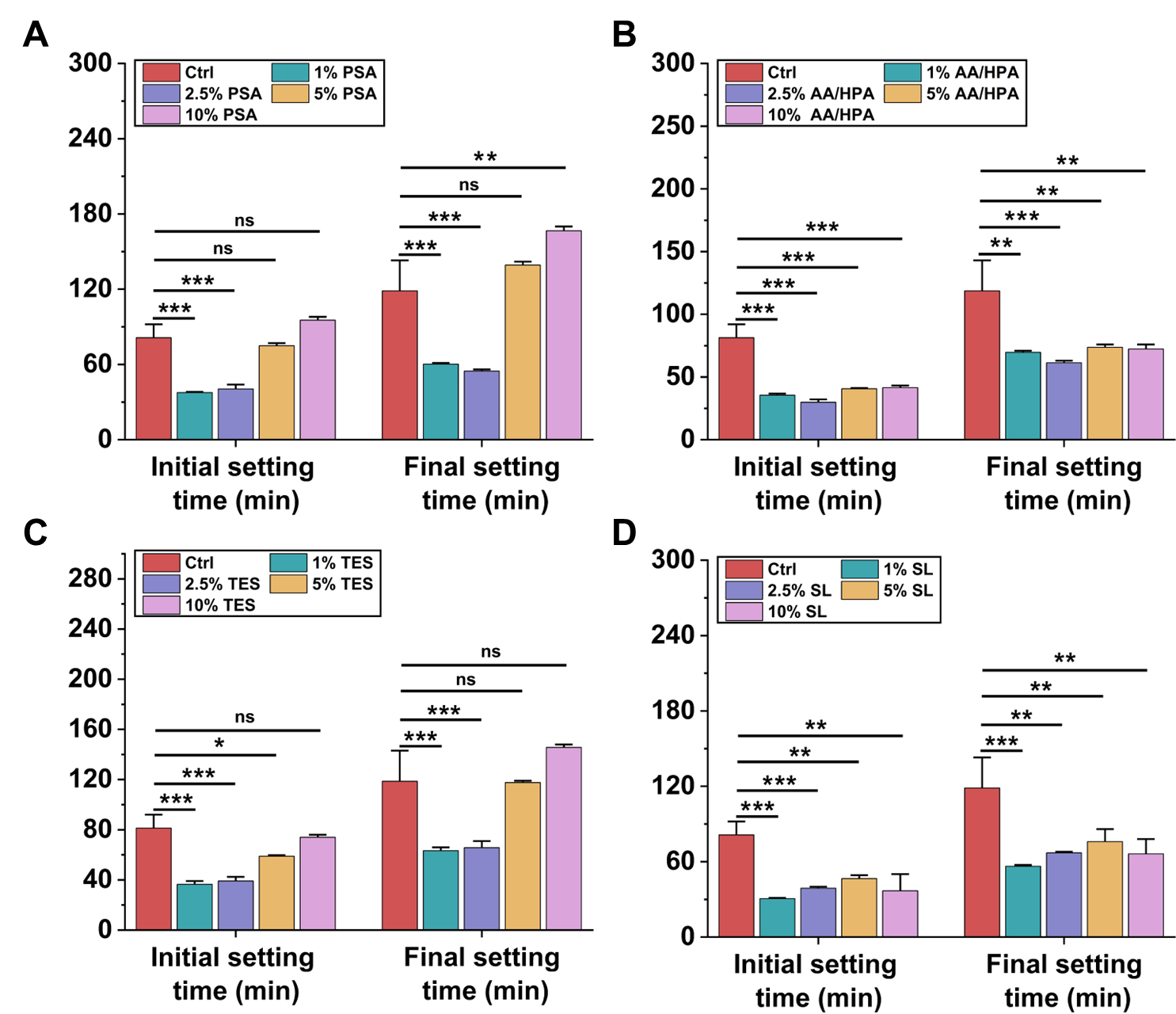
**

**Fig. S7.** Initial and final setting times of brushite cements (liquid phase: 0.55 M AIC + different wt.% amounts of superplasticizer, powder phase: β-TCP + MCPM) modified with different superplasticizers at an L/P of 0.25 mL/g. (A) PSA, (B) AA/HPA, (C) TES, and (D) SL. Error bars represent standard deviations obtained by three independent repeated measurements (Ctrl: 0.55 M AIC).


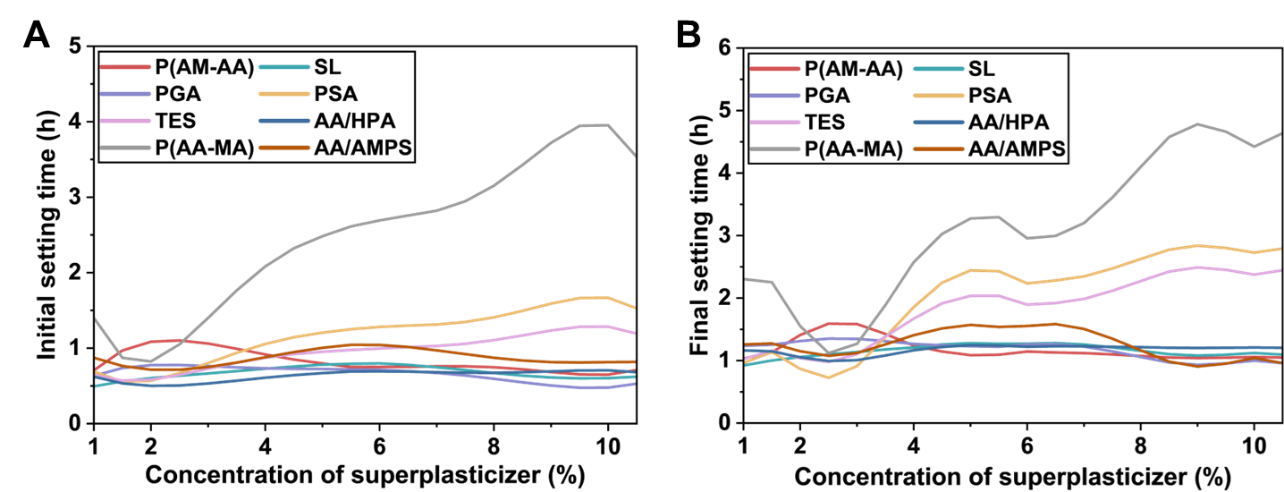


**Fig. S8.** Prediction of (A) initial setting time and (B) final setting time influenced by the superplasticizer concentration using the Random Forest model.


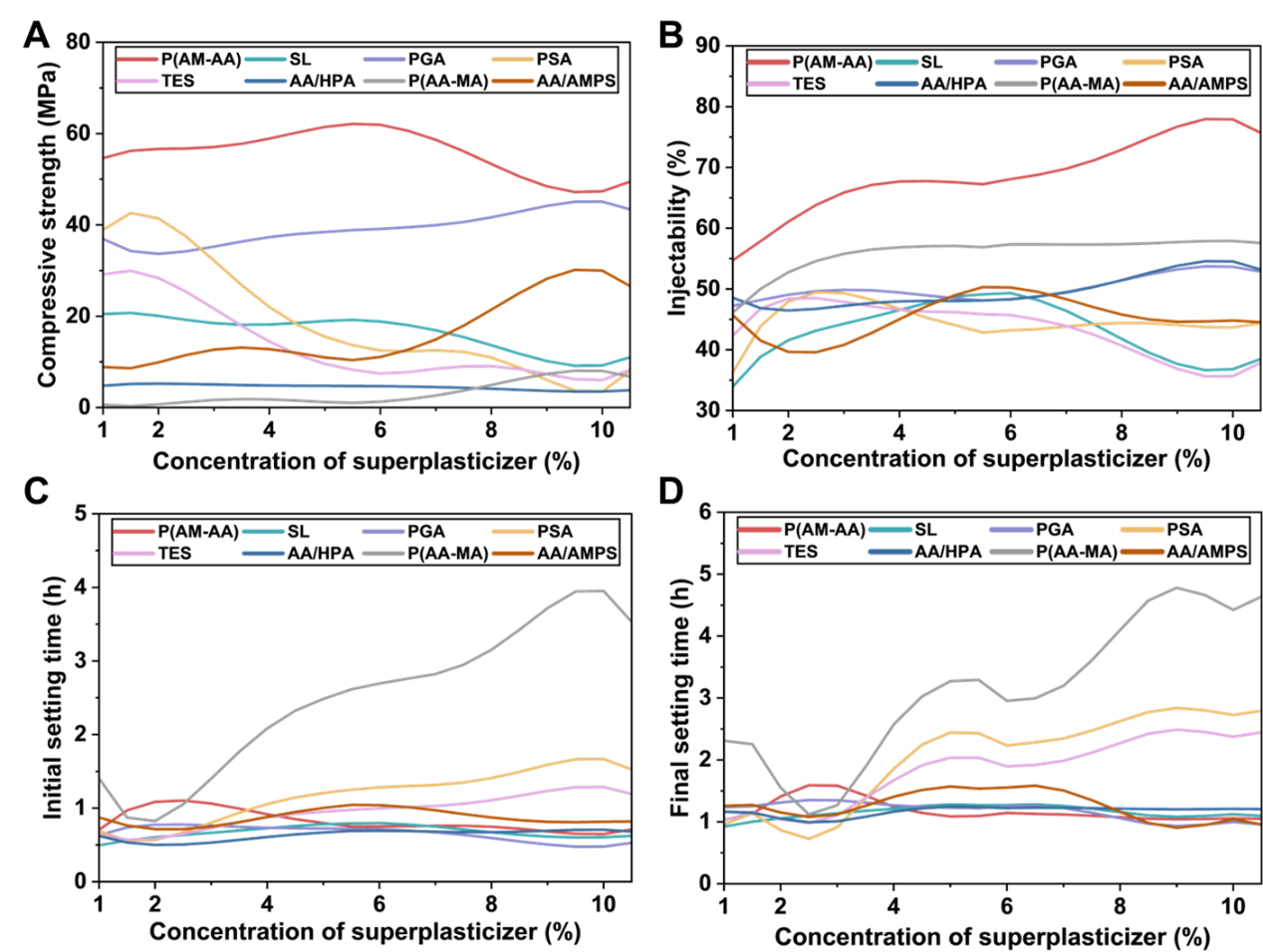


**Fig. S9.** Prediction of (A) compressive strength, (B) injectability, (C) initial setting time, and (D) final setting time influenced by the superplasticizer concentration using the Decision Tree model.


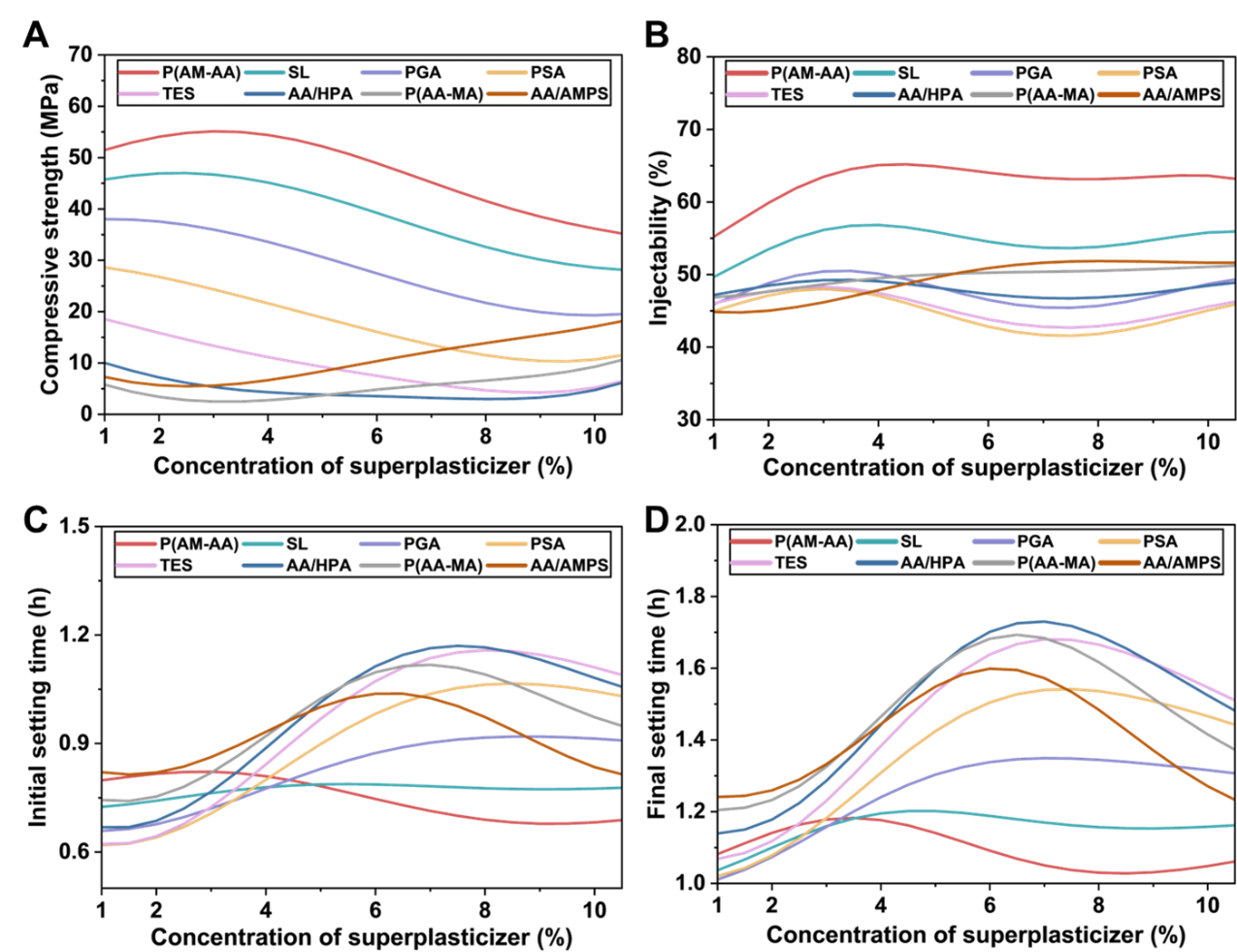


**Fig. S10.** Prediction of (A) compressive strength, (B) injectability, (C) initial setting time and, (D) final setting time influenced by the superplasticizer concentration using the Support Vector Machine model.


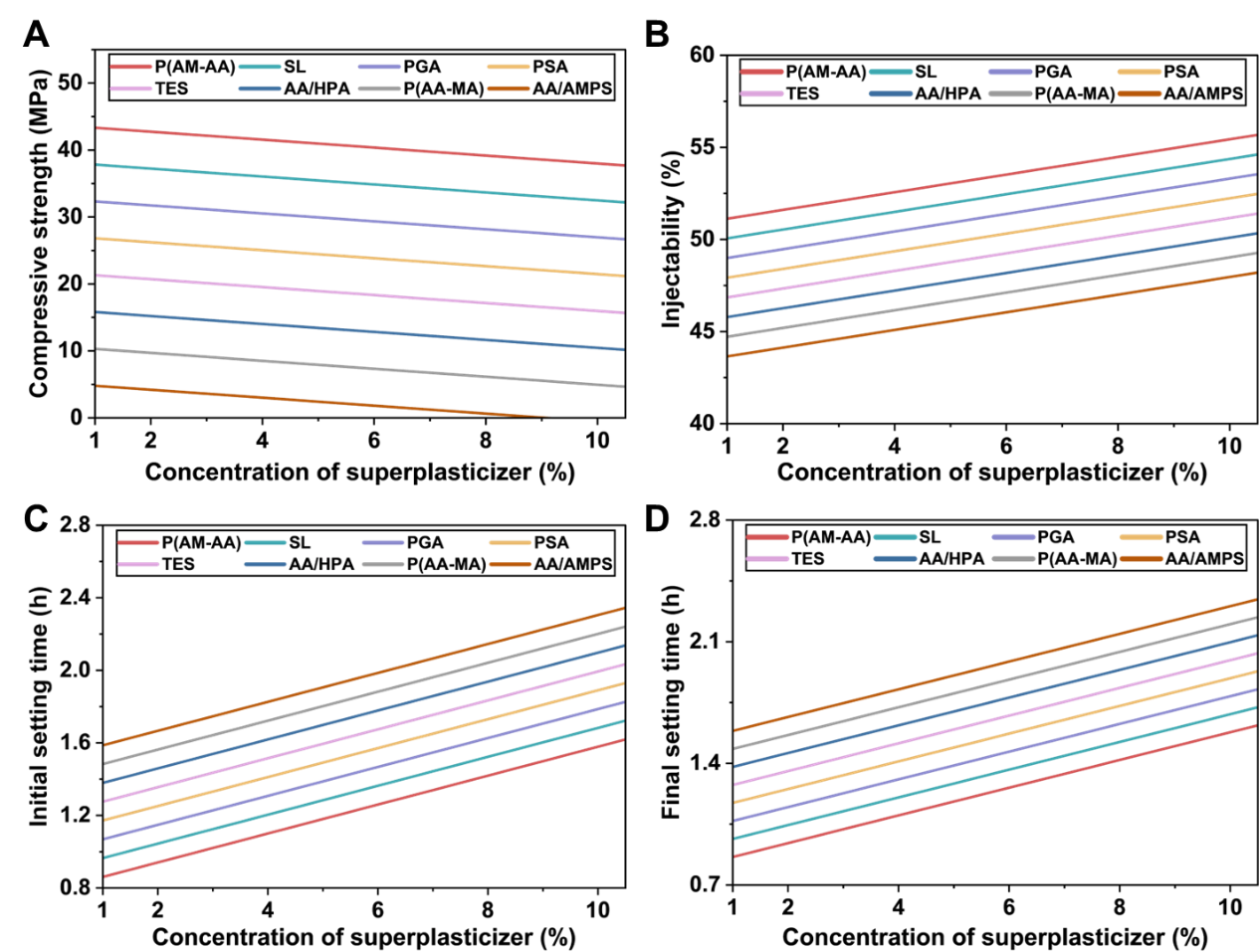


**Fig. S11.** Prediction of (A) compressive strength, (B) injectability, (C) initial setting time, and (D) final setting time influenced by the superplasticizer concentration using the Full Connect Neural Network model.


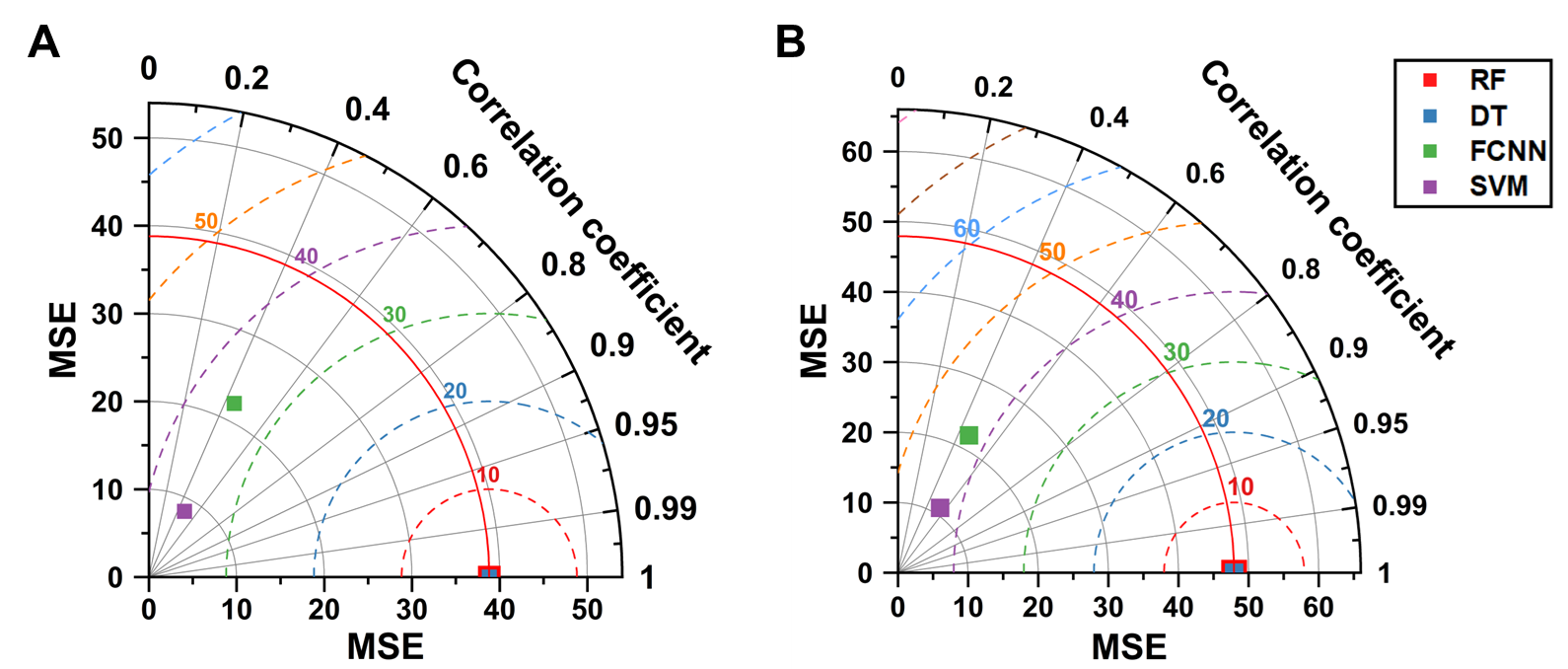


**Fig. S12.** Taylor diagrams for the error metrics of different models corresponding to training data of (A) initial setting time and (B) final setting time. [The ideal values (R^2^, MSE, RMSE) for these indices are as follows: R^2^ should ideally be 1, indicating perfect prediction accuracy, while RMSE and MSE should ideally be 0, indicating no error in the predictions].


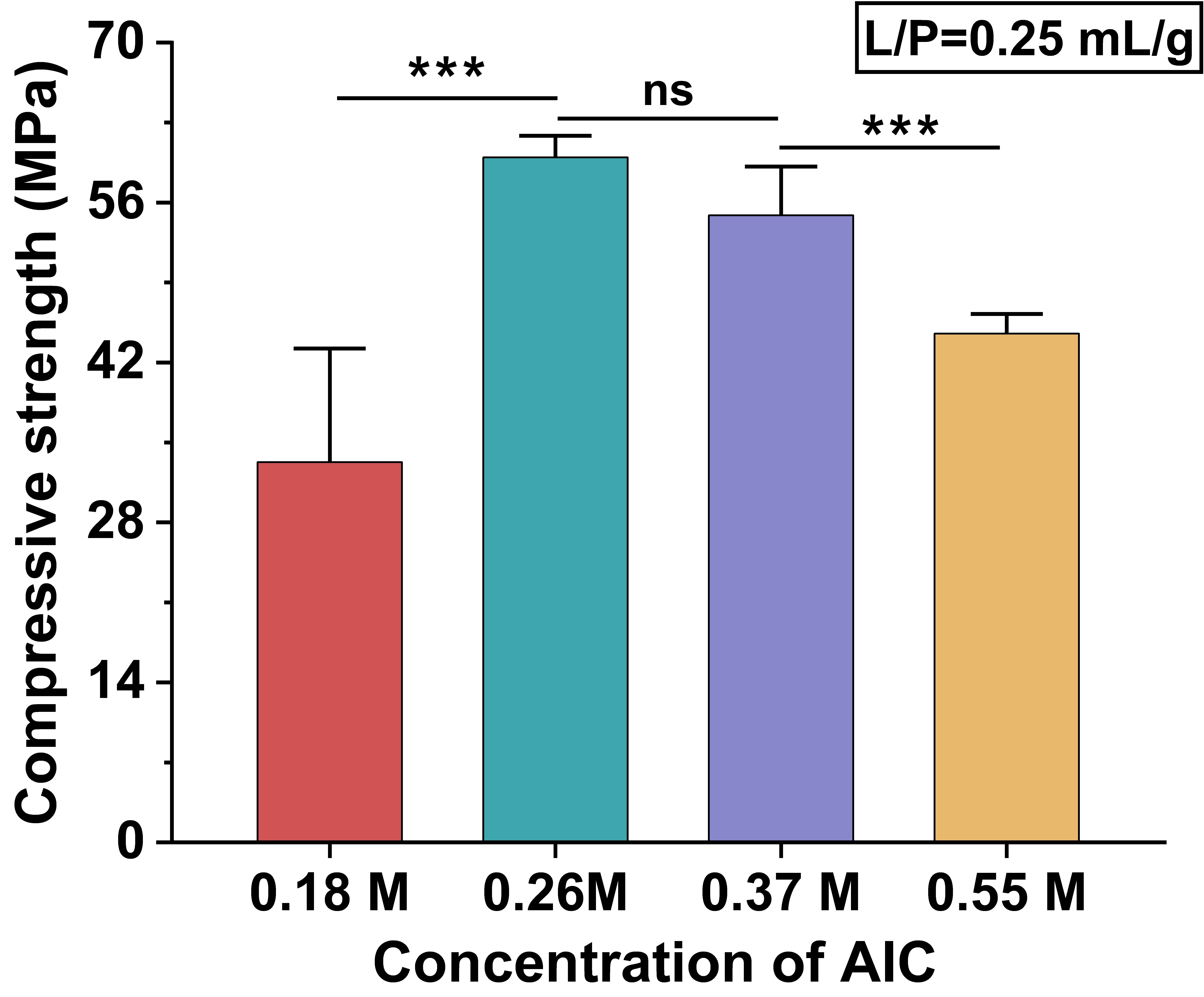


**Fig. S13.** Compressive strength of brushite cements (liquid phase: AIC, powder phase: β-TCP + MCPM) with different AIC concentrations without superplasticizer. Error bars represent standard deviations obtained by five independent repeated measurements. Statistical analysis was performed using one-way ANOVA, with significance defined as **p* < 0.05.

**
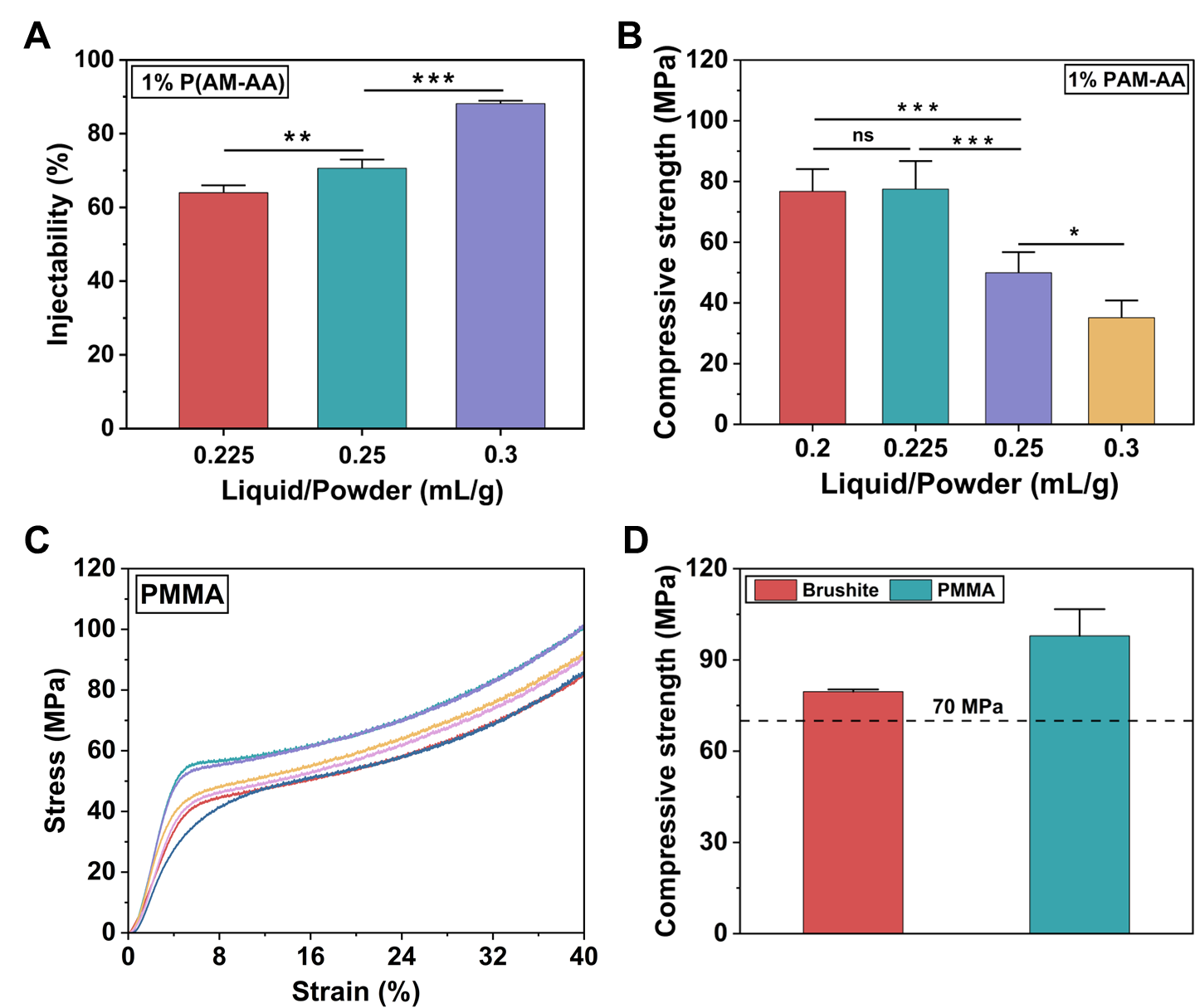
**

**Fig. S14.** (A) Injectability and (B) compressive strength of brushite cements with 1% P(AM-AA) at different L/P ratios. (C) Stress-strain curves of PMMA cements (n=6). (D) Compressive strength of brushite and PMMA cements (ISO 5833). Error bars represent standard deviations obtained by three independent repeated measurements. Statistical analysis was performed using one-way ANOVA, with significance defined as **p* < 0.05.

**
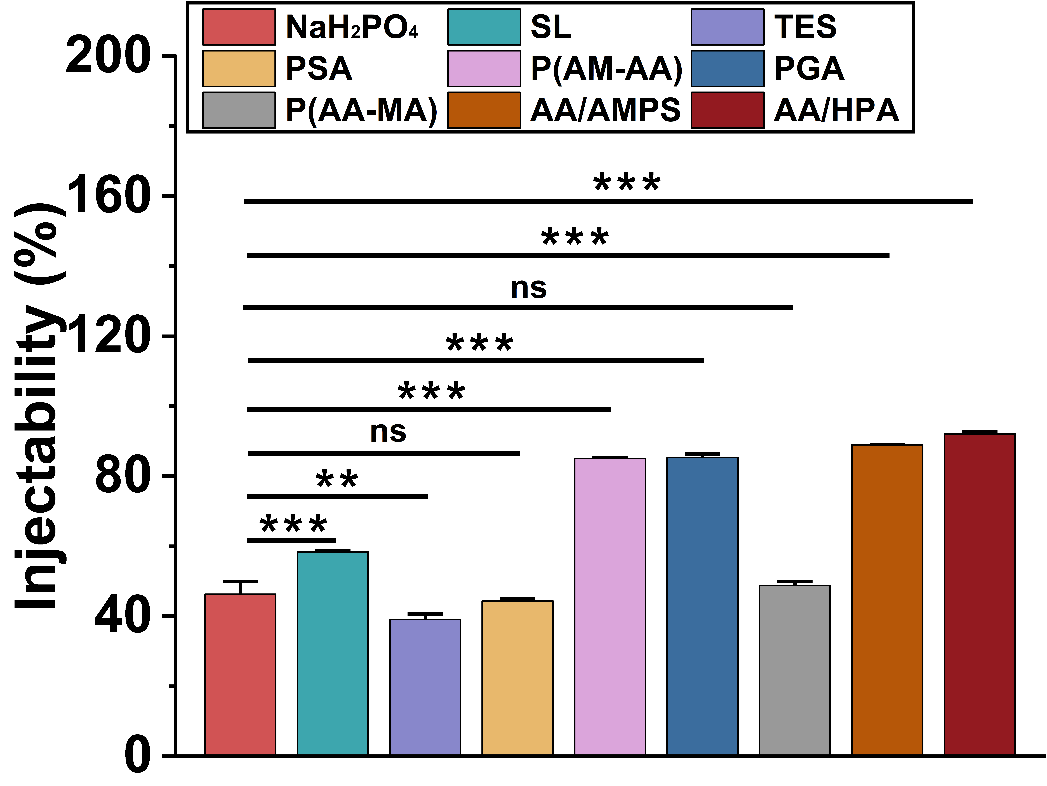
**

**Fig. S15.** Injectability of apatite cements (liquid phase: 2.5 wt.% NaH_2_PO_4_ + 5 wt.% superplasticizer, powder phase: α-TCP) modified with various superplasticizers at an L/P ratio of 0.4 mL/g. Error bars represent standard deviations obtained by three independent repeated measurements.


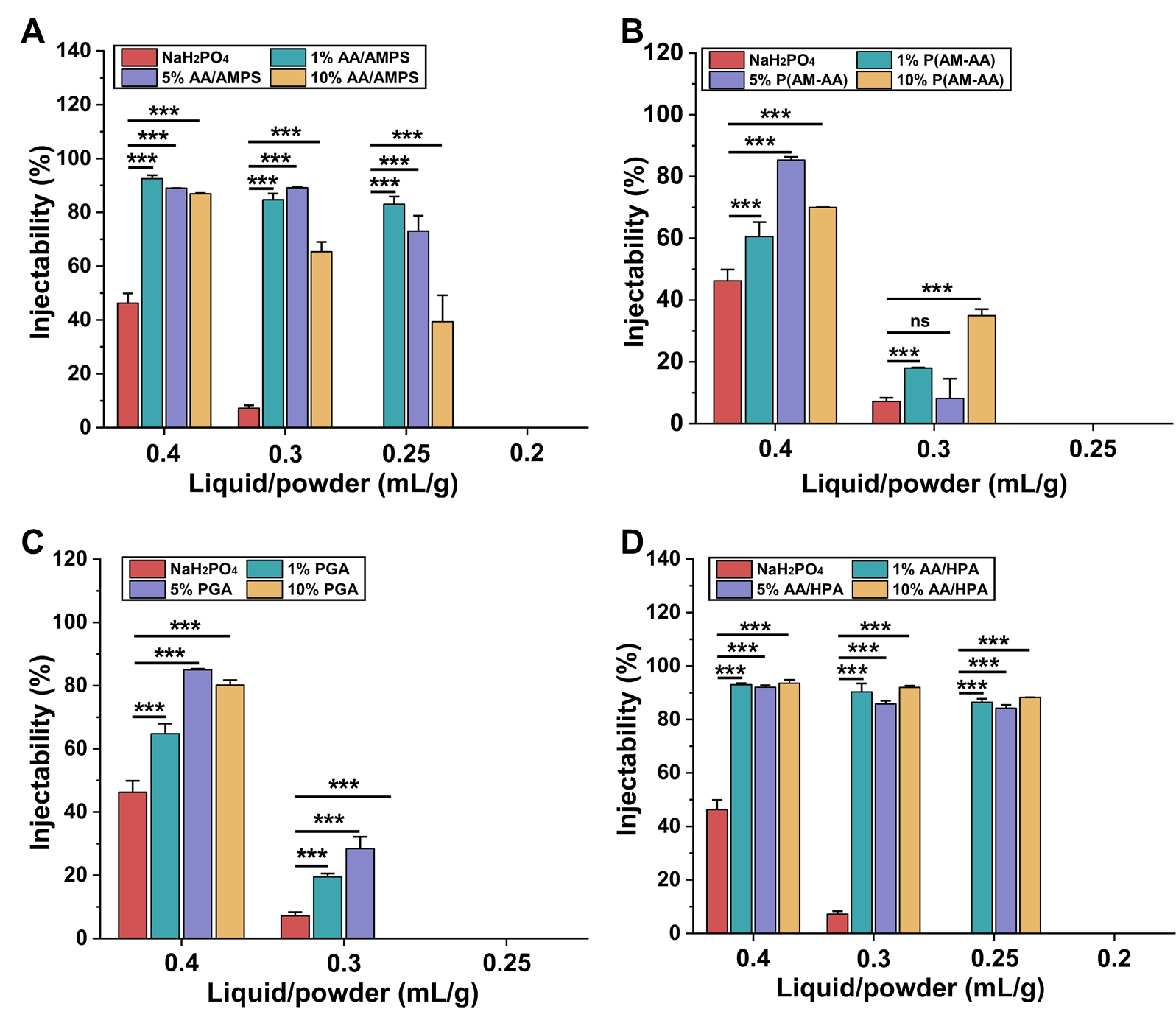


**Fig. S16.** Injectability of apatite cements (liquid phase: 2.5 wt.% NaH_2_PO_4_ + different wt.% amounts of superplasticizer, powder phase: α-TCP) modified with (A) AA/AMPS, (B) P(AM-AA), (C) PGA, and (D) AA/HPA. Error bars represent standard deviations obtained by three independent repeated measurements.


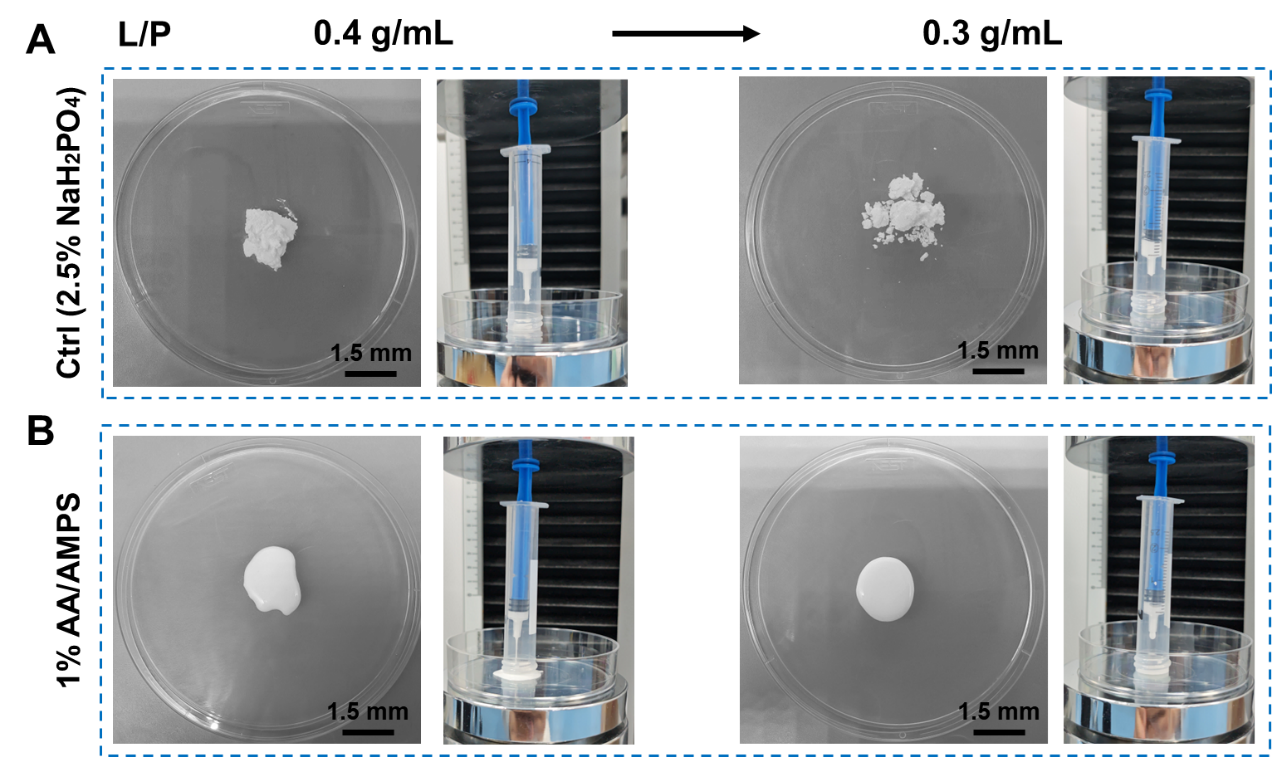


**Fig. S17.** Gross images mixed with liquid and powder phases and the injectability of apatite cement (L/P from 0.4 mL/g to 0.3 mL/g) modified with superplasticizer. Apatite cement (A) without AA/AMPS and (B) with AA/AMPS.

**
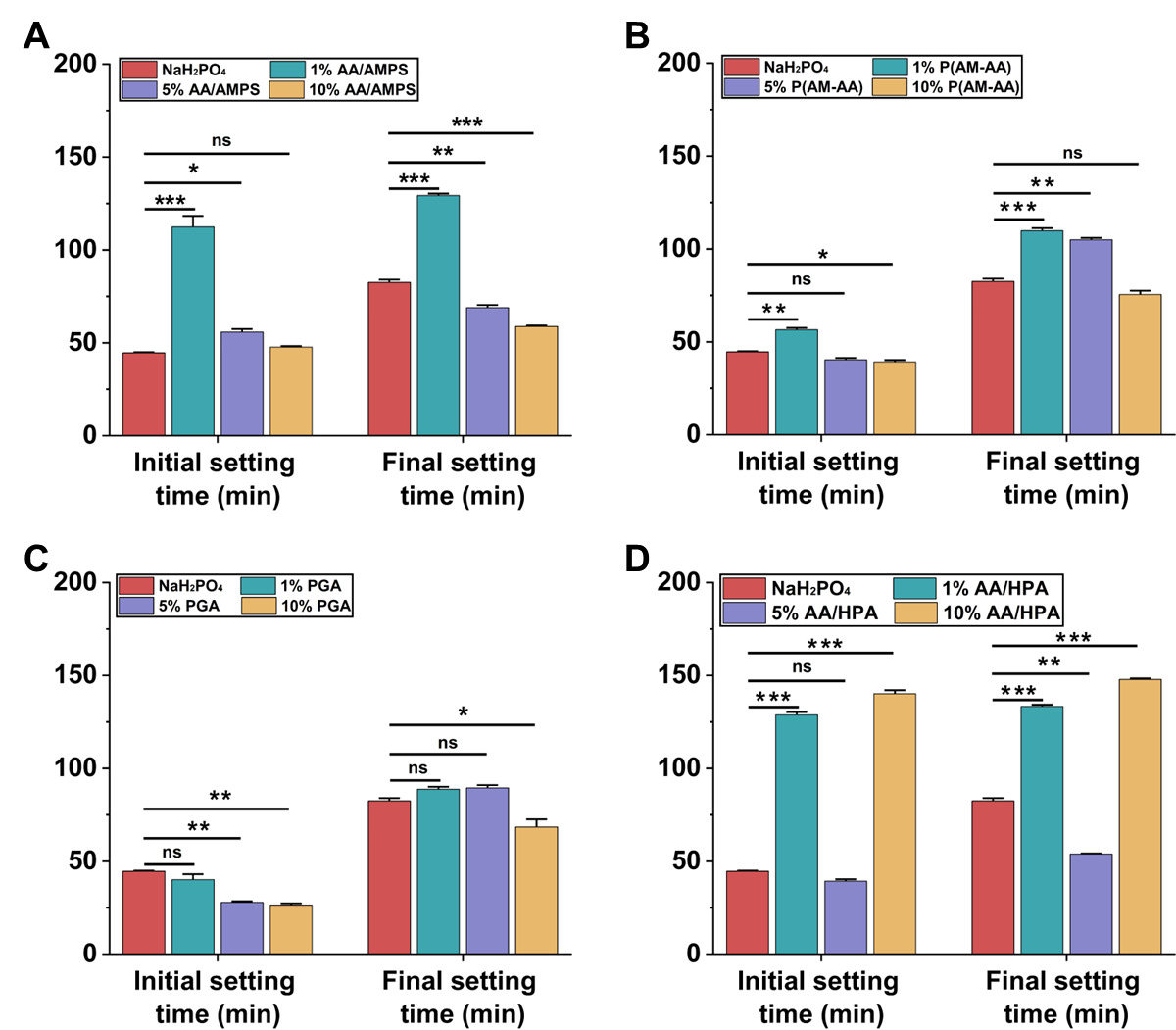
**

**Fig. S18.** Setting times of apatite cements (liquid phase: 2.5 wt.% NaH_2_PO_4_ + different wt.% amounts of superplasticizer, powder phase: α-TCP) modified by (A) AA/AMPS, (B) P(AM-AA), (C) PGA and (D) AA/HPA at an L/P of 0.4 mL/g. Error bars represent standard deviations obtained by three independent repeated measurements.

**
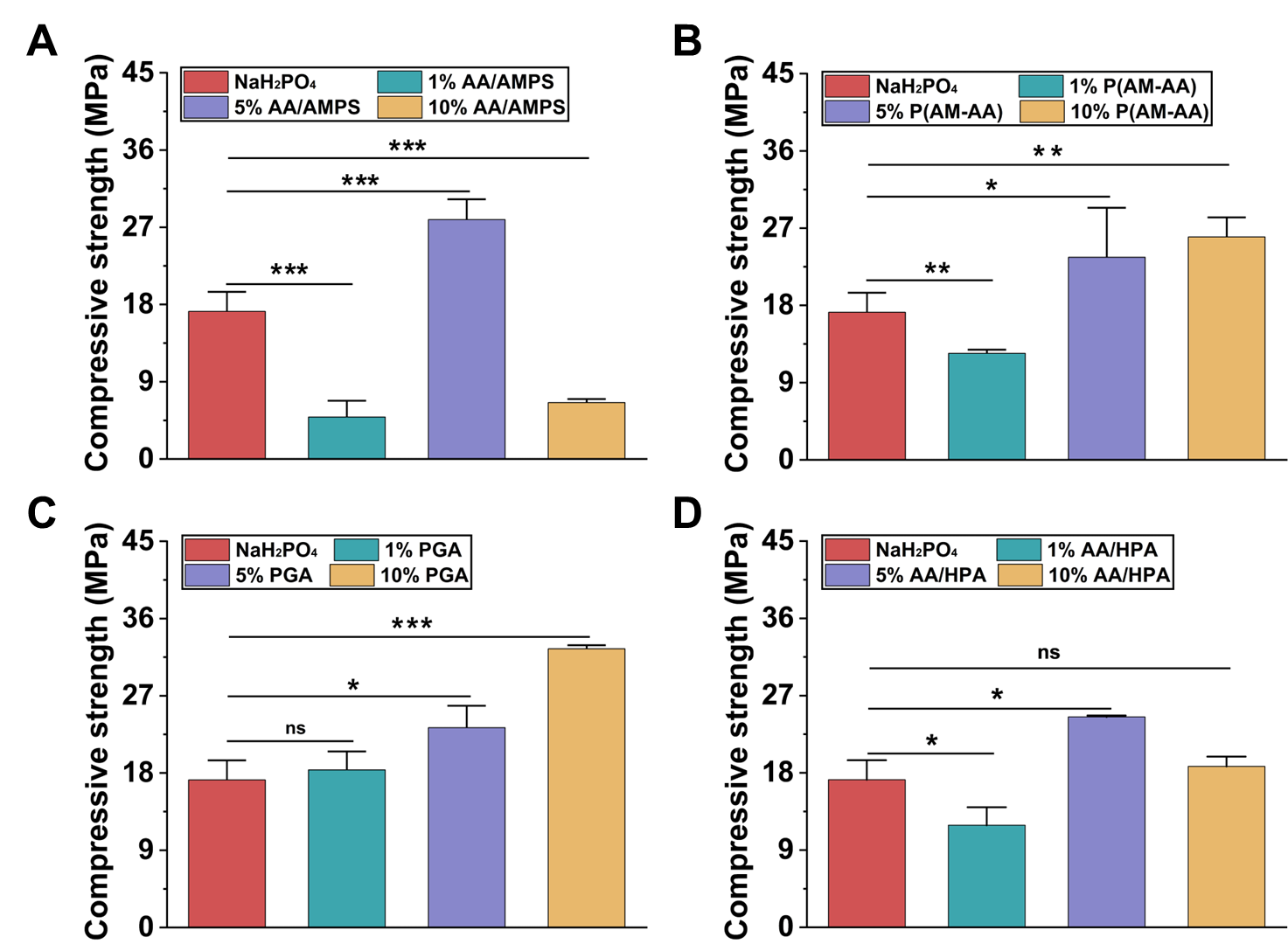
**

**Fig. S19.** Compressive strength of apatite cements (liquid phase: 2.5 wt.% NaH_2_PO_4_ + different wt.% amounts of superplasticizer, powder phase: α-TCP) modified by (A) AA/AMPS, (B) P(AM-AA), (C) PGA, and (D) AA/HPA at an L/P of 0.4 mL/g. Error bars represent standard deviations obtained by five independent repeated measurements.

**
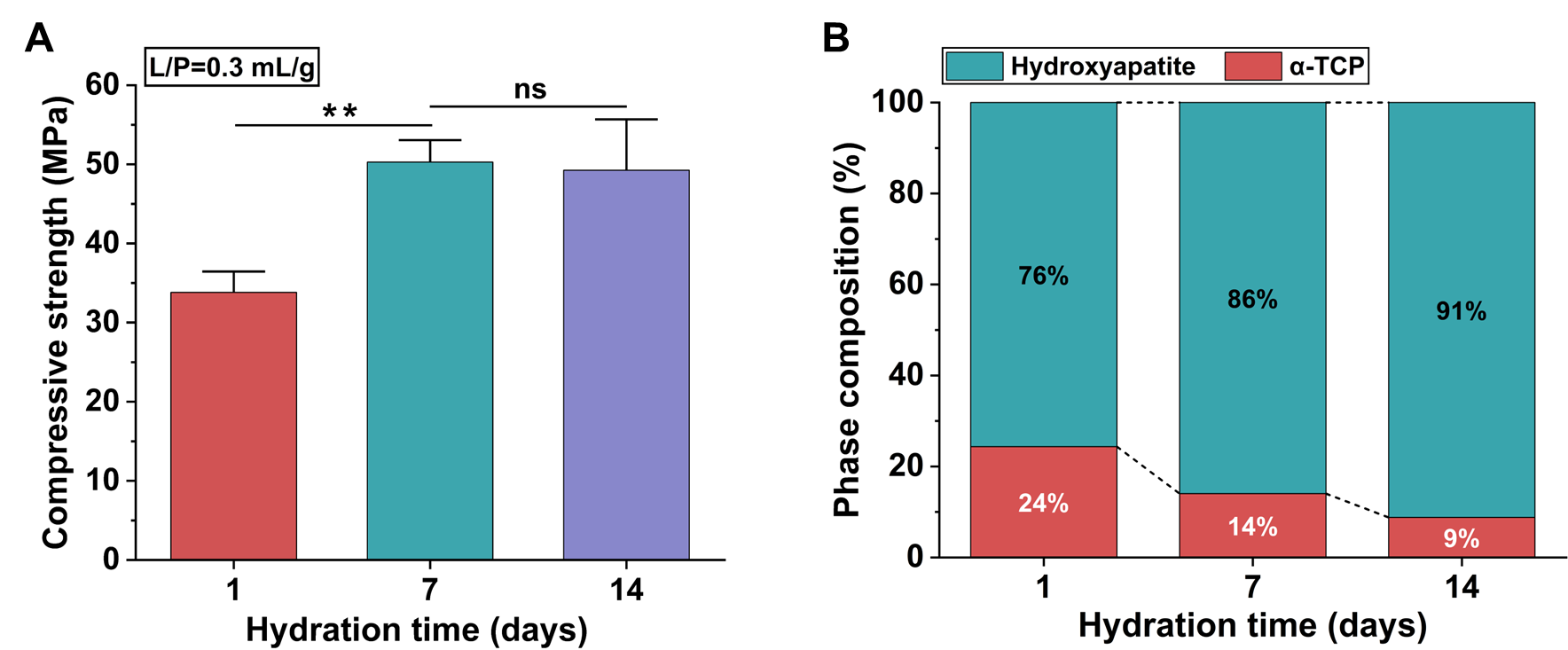
**

**Fig. S20.** (A) Compressive strength of apatite cements modified with 5 wt.% AA/AMPS at different hydration times (1, 7, and 14 days). (B) The phase composition of apatite cements modified by AA/AMPS after different hydration times. Error bars represent the standard deviation obtained by three independent repeated measurements. Statistical analysis was performed using one-way ANOVA, with significance defined as **p* < 0.05.


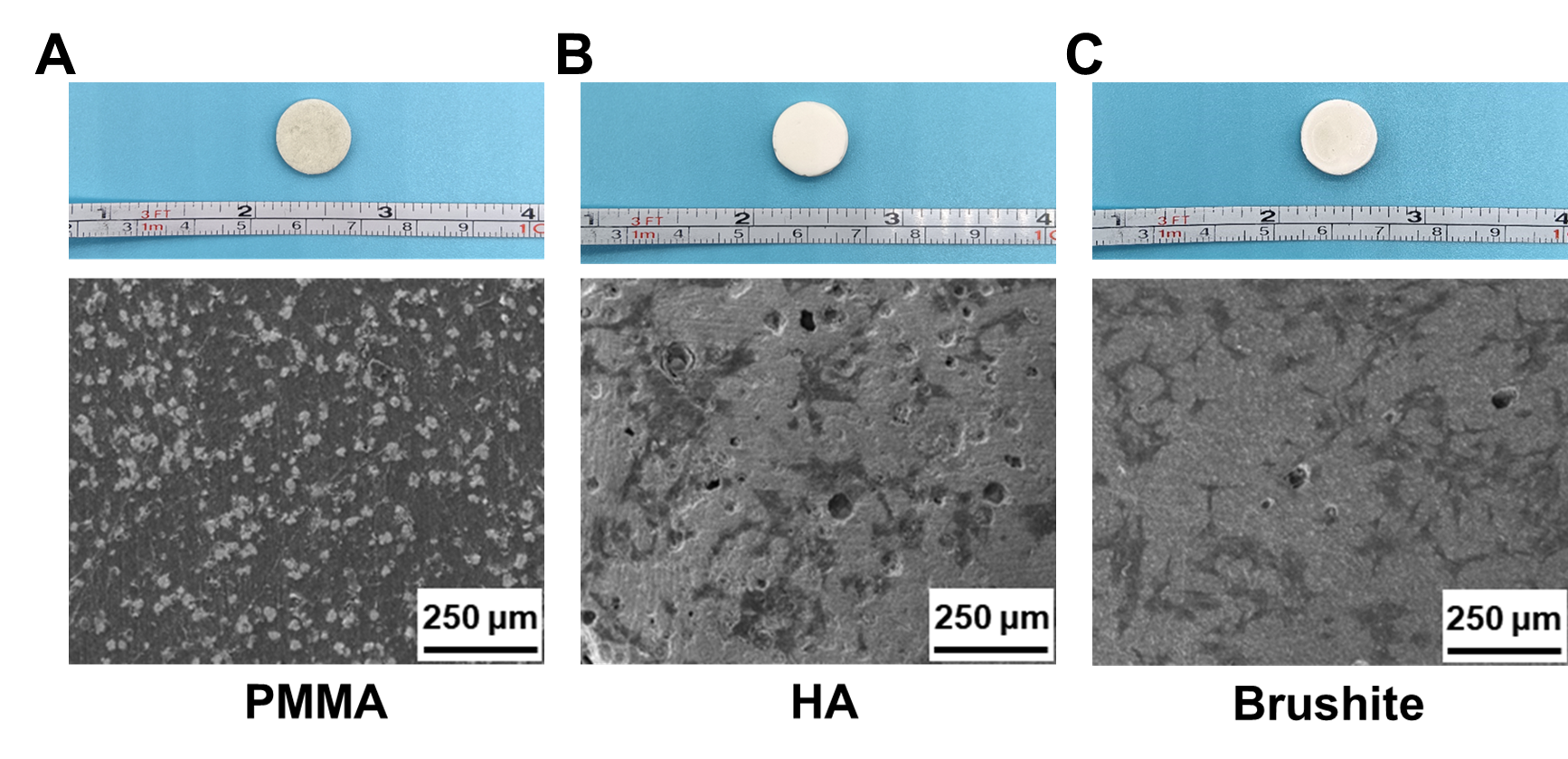


**Fig. S21.** SEM images of BMSCs cultured on (A) PMMA, (B) HA, and (C) brushite cements.

**
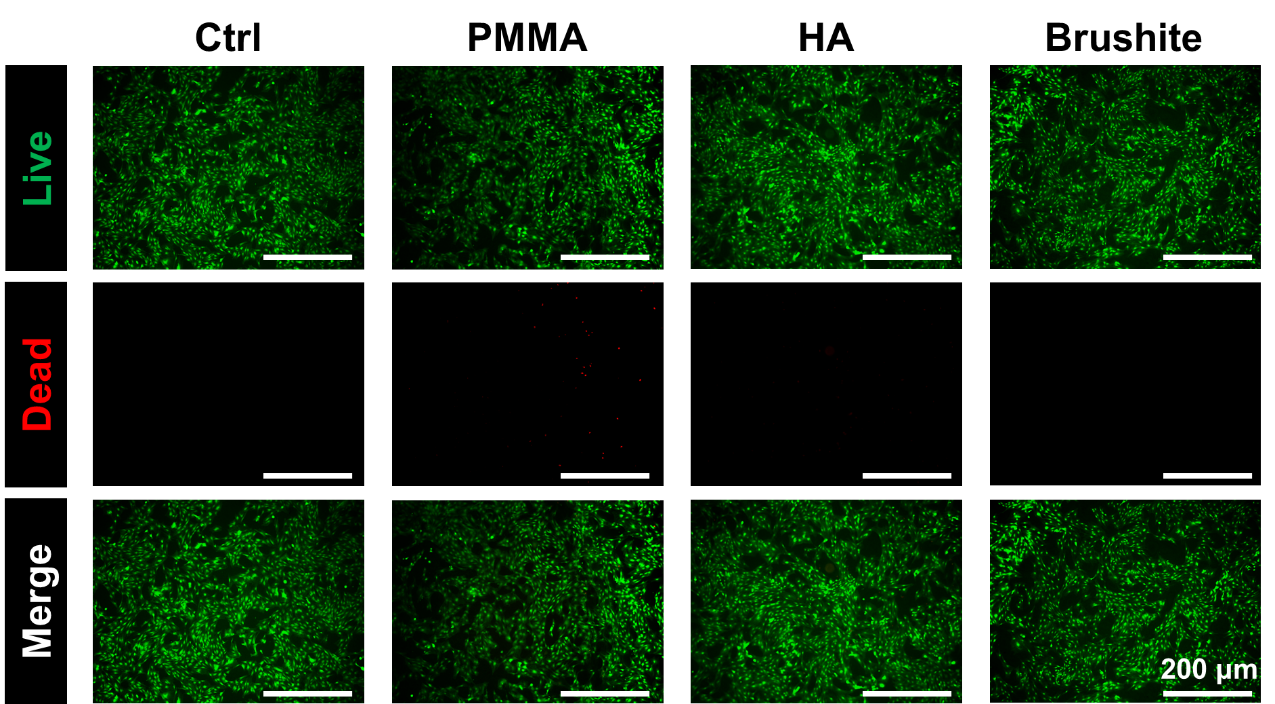
**

**Fig. S22.** Live-dead staining of BMSCs cultured for 3 days with extracts of PMMA, HA, and brushite cements.

**
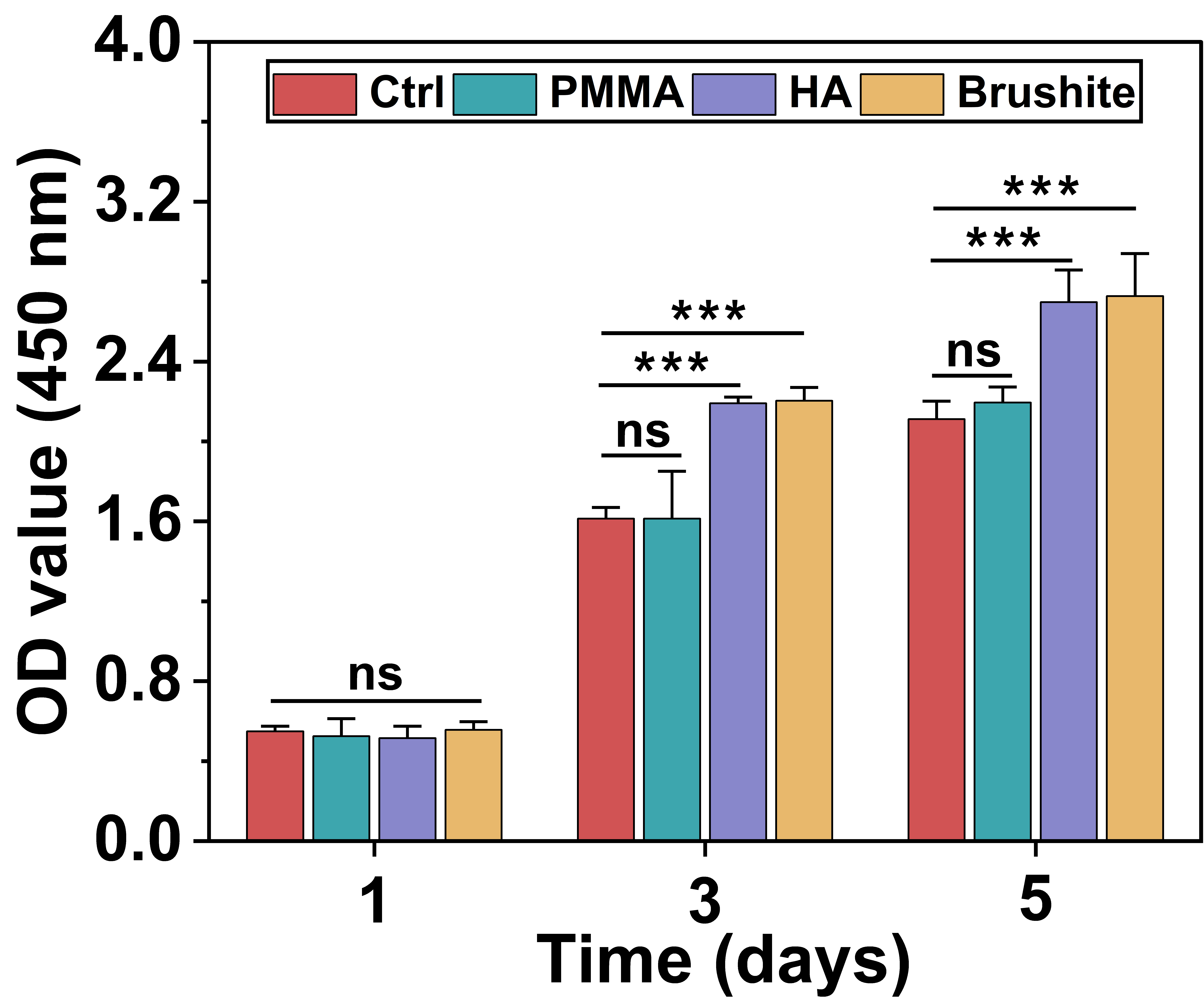
**

**Fig. S23.** Proliferation of BMSCs cultured for 1, 3, and 5 days in extracts of PMMA, HA, and brushite cements. Error bars represent standard deviations obtained by five independent repeated measurements. Statistical analysis was performed using one-way ANOVA, with significance defined as **p* < 0.05.

**
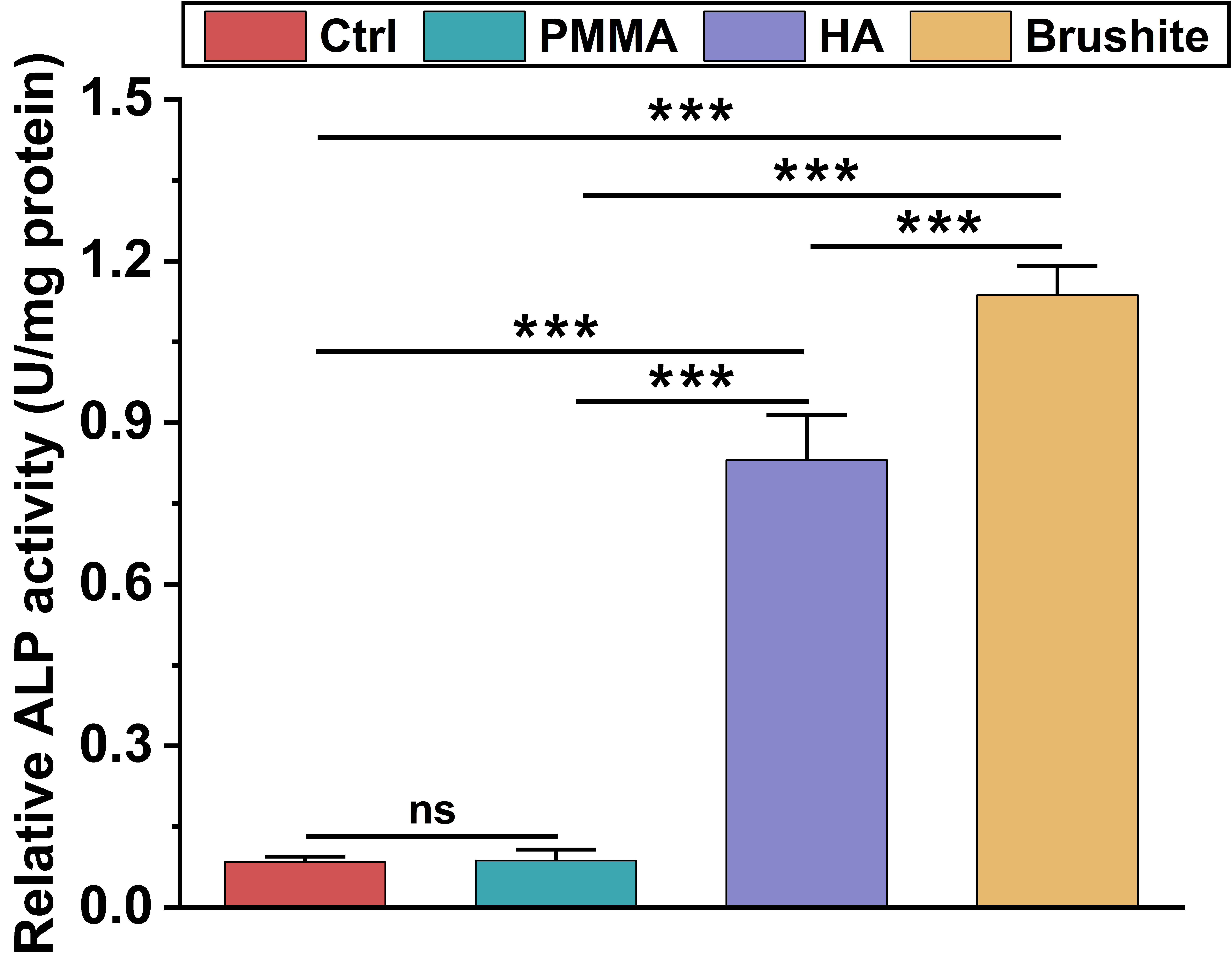
**

**Fig. S24.** Alkaline phosphatase (ALP) staining of BMSCs cultured for 7 days with extracts of PMMA, HA, and brushite cements. Error bars represent standard deviations obtained by three independent repeated measurements. Statistical analysis was performed using one-way ANOVA, with significance defined as **p* < 0.05.


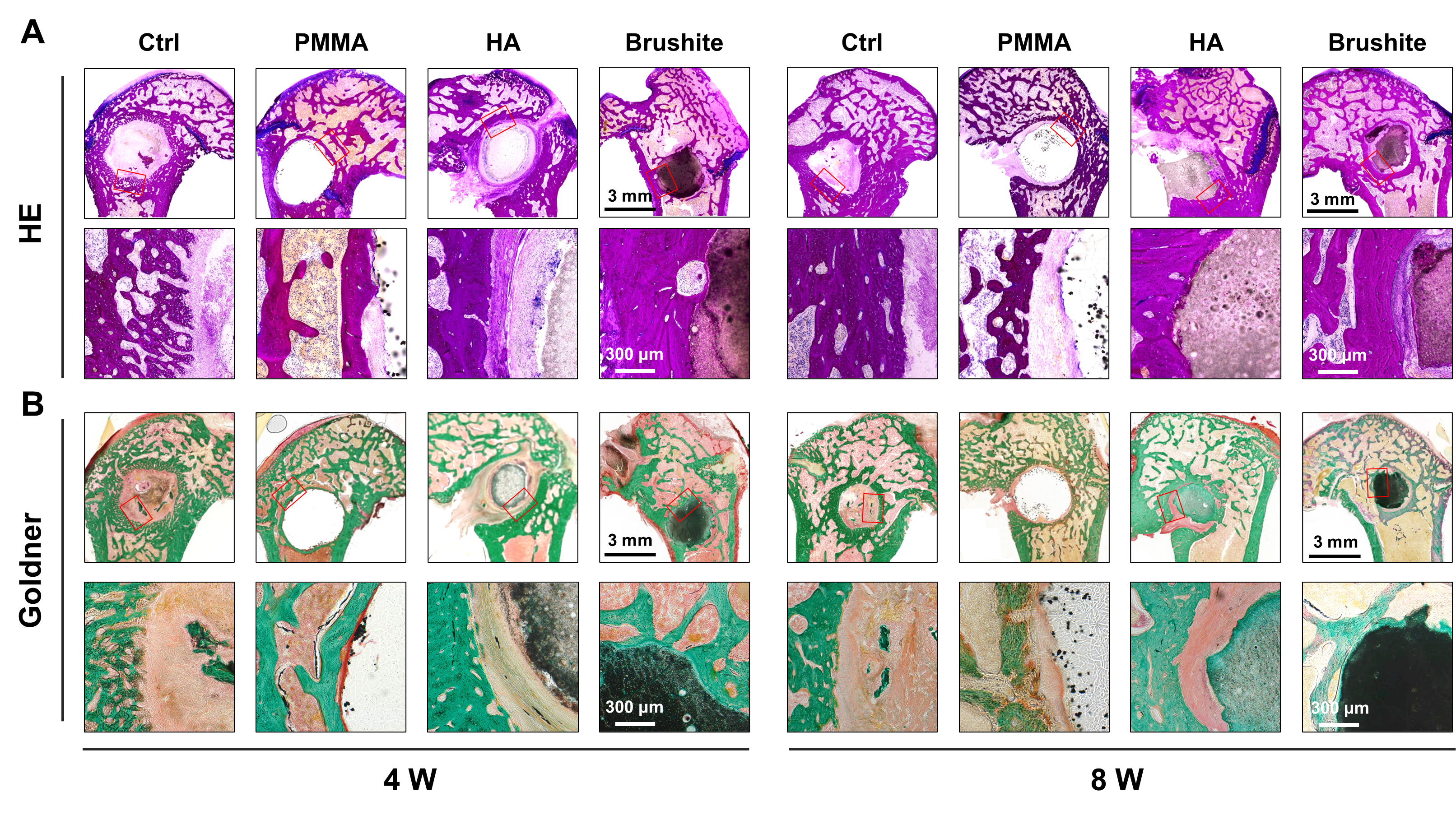


**Fig. S25.** Histological staining of hard tissue sections. (A) H&E staining of the femur of a hard tissue section after 4 and 8 weeks. (B) Goldner staining of the femur of a hard tissue section after 4 and 8 weeks.
